# Global fire history of grassland biomes

**DOI:** 10.1002/ece3.4394

**Published:** 2018-08-10

**Authors:** Berangere A. Leys, Jennifer R. Marlon, Charles Umbanhowar, Boris Vannière

**Affiliations:** ^1^ Department of Geography Kansas State University Manhattan Kansas; ^2^ Chrono‐environnement UMR6249 CNRS Université Bourgogne Franche‐Comté Besançon France; ^3^ School of Forestry and Environmental Studies Yale University New Haven Connecticut; ^4^ Departments of Biology and Environmental Studies Saint Olaf College Northfield Minnesota

**Keywords:** biome classifications, charcoal, fire regime, global biomass burning, grasslands, Holocene

## Abstract

Grasslands are globally extensive; they exist in many different climates, at high and low elevations, on nutrient‐rich and nutrient‐poor soils. Grassland distributions today are closely linked to human activities, herbivores, and fire, but many have been converted to urban areas, forests, or agriculture fields. Roughly 80% of fires globally occur in grasslands each year, making fire a critical process in grassland dynamics. Yet, little is known about the long‐term history of fire in grasslands. Here, we analyze sedimentary archives to reconstruct grassland fire histories during the Holocene. Given that grassland locations change over time, we compare several charcoal‐based fire reconstructions based on alternative classification schemes: (a) sites from modern grassland locations; (b) sites that were likely grasslands during the mid‐Holocene; and (c) sites based on author‐derived classifications. We also compare fire histories from grassland sites, forested sites, and all sites globally over the past 12,000 years. Forested versus grassland sites show different trends: grassland burning increased from the early to mid‐Holocene, reaching a maximum about 8000–6000 years ago, and subsequently declined, reaching a minimum around 4000 years ago. In contrast, biomass burning in forests increased during the Holocene until about 2000 years ago. Continental grassland fire history reconstructions show opposing Holocene trends in North versus South America, whereas grassland burning in Australia was highly variable in the early Holocene and much more stable after the mid‐Holocene. The sharp differences in continental as well as forest versus grassland Holocene fire history trajectories have important implications for our understanding of global biomass burning and its emissions, the global carbon cycle, biodiversity, conservation, and land management.

## 1 INTRODUCTION

**Figure 1:**
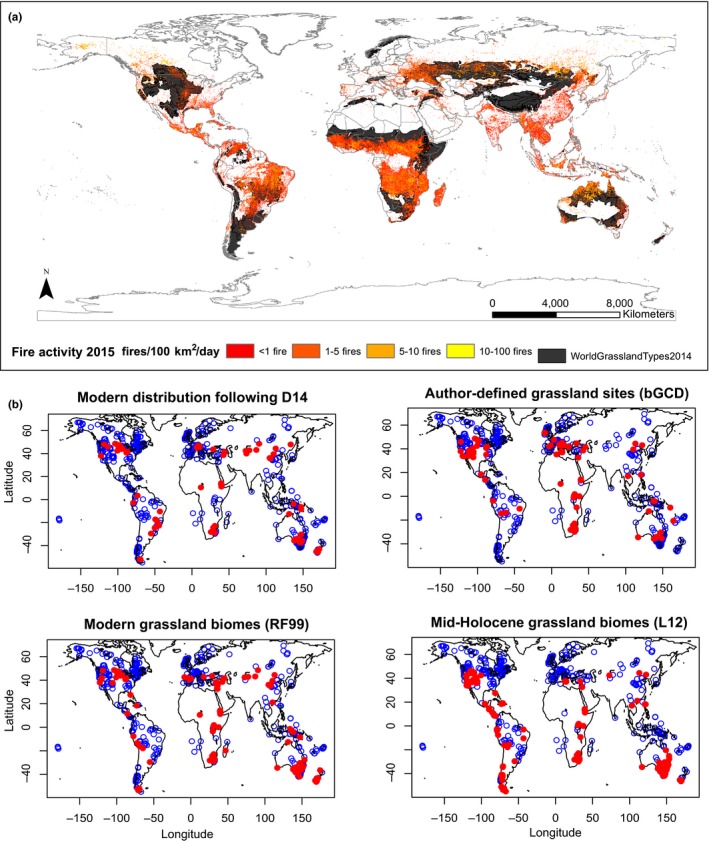
(a) Fire activity for 2015 (NASA Observatory). White pixels represent the high end of the fire count, with as many as 100 fires/1000 km²/day, yellow pixels represent as many as 10 fires/1000 km²/day, orange pixels represent as many as 5 fires/1000 km²/day, and red pixels represent as few as 1 fires/1000 km²/day. Black areas correspond to grassland distribution from Dixon et al., 2014. (b**)** Site selections by biome classifications (red dots) from the Global Charcoal Database (blue dots corresponding to all the available sites in the GCD): grassland and savanna biomes following L12 classification; all the sites overlapping the current grassland distribution published by Dixon et al. (2014); grassland and savanna biomes following Ramankutty and Foley (1999) classification (RF99); and grassland biomes as described by contributors of the GCD (bGCD; http://www.paleofire.org) [Colour figure can be viewed at http://wileyonlinelibrary.com]

Grasslands, broadly defined, occur across a wide range of environmental conditions, from the tropics to the Arctic, from sea level to mountain tops, in arid and humid areas, and in locations with thin, poor soils to deep nutrient‐rich soils (Figure 1a; Dixon et al., 2014; Still, Berry, Collatz, & DeFries, 2003). Grasslands are a key component of the Earth system, playing critical roles in cycling nutrients, water, and carbon, and in sustaining biodiversity, wild game, and livestock. While grassland ecosystems vary widely in composition and structure (including shrublands, steppe, and savanna, for example), most are in decline today as forests, croplands, agricultural fields, and urbanization all expand. Grasslands globally have been reduced in their areal extent by 40% since the Industrial Era (Murray, White, & Rohweder, 2000; White, Murray, Rohweder, Prince, & Thompson, 2000), causing losses in the ecosystem services they provide, such as carbon storage in soils and biodiversity (Murray et al., 2000; Veldman et al., 2015). Despite widespread recognition of their importance and varied attempts to protect grasslands around the world, managing and conserving their essential processes remains a significant challenge.

Most grasslands occur in regions where tree cover is limited by edaphic or climate conditions, or by disturbances such as fire, herbivory, or flooding. The particular factors that control the distribution of grasslands across regions and continents are varied and debated, but fire is a consistently strong predictor (Staver, Archibald, & Levin, 2011). Across a wide range of climatic and soil conditions, frequent fires and herbivory limit the abundance of trees and shrubs, promote herbaceous productivity, consume dead plant material, return nutrients to the soil, stimulate reproduction, and maintain plant diversity (Lehmann et al., 2014; Nayak, Vaidyanathan, & Krishnaswamy, 2014; Veldman et al., 2014, 2015). Frequent fires and herbivory are particularly critical processes where precipitation and soil nutrient availability are sufficient to permit forest development (Bond, 2008; Bond & Keeley, 2005; Staver et al., 2011). Moreover, grassland ecosystems represent over one‐third of Earth's vegetation cover (Table 1), but roughly 80% of global fire takes place in these systems each year (Figure 1a, Mouillot & Field, 2005), representing about 40% of the gross global carbon dioxide emissions (Murray et al., 2000).

**Table 1:** Selected biomes and number of sites for the four classifications: RF99, D14, L12, and GCD. Total number of charcoal records in the selected biomes, and the number and percentages of sites by continent. Grasslands areal extent (in percentages) are calculated from Dixon et al. (2014) for each continent based on the area of grassland in each continent divided by the total area of grasslands globally. Colors refer to the sampling density of fire records based on the percentage of sites with charcoal data either above (red) or below (blue) the percentage of grassland area by continent

Site selection	Source	Selected biome types	Sites	Total	North America	South America	Eurasia	Africa	Oceania
Modern grassland biomes (RF99)	Ramankutty and Foley (1999)	Savanna, Grassland/Steppe, Open shrubland	Number	157	21	12	44	23	57
			%		13	8	28	15	36
Modern distribution (D14)	Dixon et al. (2014)	Sites overlapping the current distribution of grasslands following Dixon et al. (2014)	Number	108	15	11	41	15	26
			%		14	10	38	14	24
Mid‐Holocene grassland biomes (L12)	Levavasseur et al. (2012)	Grassland and dry shrubland, Savanna and dry woodlands	Number	262	39	46	26	23	128
			%		15	17	10	9	49
Author‐defined grassland sites (bGCD)	Biome classification from contributors	Temperate xerophytic shrubland, Temperate deciduous broadleaf savanna, Temperate grassland, Desert, Temperate xerophytic woodland and shrubland, Tropical xerophytic shrubland, Tropical savanna	Number	103	22	7	39	20	15
			%		21	7	38	19	15
			% surface grassland	100	12	16	30	30	12

Despite the critical roles of fire in grasslands, we know relatively little about how burning varied in these ecosystems before the advent of satellite records. The vast majority of studies focusing on grasslands have provided information based on modern ecological data, or on historical or paleoecological reconstructions of changes within a single watershed or small region (Heisler, Briggs, & Knapp, 2003; Morgan, 1999; Robin et al., 2018; Zhijun, Jiquan, & Xingpeng, 2009). The lack of long‐term data about the effects of rising greenhouse gases, changes in temperature, and hydrology on biomass in grasslands, in particular, poses a significant obstacle to understanding both past dynamics and potential future trajectories of grassland ecosystems.

A variety of global fire history reconstructions since the Last Glacial Maximum (LGM; about 21,000 years ago) have been produced using charcoal‐based sediment records (Daniau et al., 2012; Marlon et al., 2016; Power et al., 2008). Such studies revealed an increase in biomass burning since the LGM, particularly during deglaciation (Power et al., 2008). Comparisons of global biomass burning trends with temperature and precipitation simulations since the LGM suggest that rising temperatures are largely responsible for the increased biomass burning (Daniau et al., 2012). More detailed analyses of global and regional biomass burning, including data from both forests and grasslands, show substantial centennial and millennial‐scale variability (Marlon et al., 2013; Vannière et al. 2011). However, forest and grassland fire history reconstructions were treated identically in these studies, without consideration of how fire in each biome type might register differently in charcoal records, or of how changing grassland distributions might alter fire history reconstructions. Developing a better understanding of the fire history of grasslands, and what they can and cannot tell us about the controls of fire in these ecosystems is thus a critical next step in advancing scientific knowledge of how fire regimes in grasslands have evolved over centuries and millennia. Particular questions that long‐term fire history data can potentially provide insights into focus on which aspects of fire regimes are most important for driving shifts in grassland biomass burning, and how tools like prescribed burning can be used most effectively to restore or maintain grassland services and biodiversity.

In this study, we address three research questions that draw on long‐term fire history data from locations that are considered grasslands using alternative classification schemes. Specifically, we consider (a) how has biomass burning inferred from charcoal data varied on centennial and millennial scales in grasslands; (b) how trends in biomass burning differ among grassland types and how sensitive these trends are to modern versus mid‐Holocene grassland definitions; and (c) what future research is needed to advance our understanding of fire history in grasslands. In order to provide context for the analyses, we first review the past and present role of fire in maintaining and shaping grasslands, and discuss the use and utility of different proxies for reconstructing past fires. Second, we examine global fire history from 121 “grassland” sites based on charcoal records available in the Global Charcoal Database (GCD; http://www.paleofire.org) subdivided based on four different biome classifications of grasslands. We discuss the implications of the findings for global change research, conservation, and management, and identify questions emerging from the fire history of grassland sites as compared with forested sites. At last, we present directions for future research that can improve our understanding of fire history in grasslands.

## 2 PAST AND PRESENT ROLE OF FIRE

Fire is a primary disturbance agent that maintains many grassland landscapes (Figure 1a, e.g., Brown et al., 2005a; Hoetzel, Dupont, Schefuß, Rommerskirchen, & Wefer, 2013; Jacobs & Scholeder, 2002; Scheiter et al., 2012). Most grassland fires occur in Africa and Australia during dry winters (Hély & Alleaume, 2006), but extensive fires also occur in the Americas, Asia, and Europe (Figure 1a, van der Werf et al., 2006). The rapid global agricultural expansion that occurred in the 19th and 20th centuries destroyed many grasslands; in many that remained, fire was effectively removed through landscape fragmentation, the reduction in fuels with grazing, and eventually through direct suppression of fire. Today, fire regimes remain heavily modified by human activities in most ecosystems, perhaps most notably in the tropics, where fire is still being used to convert forest to pasture (Nepstad et al., 1999). Invasive grasses facilitate this process, promoting more open, flammable communities where forest and woodlands used to exist (Balch, Bradley, D'antonio, & Gómez‐Dans, 2013; Cochrane et al., 1999). To understand how fire regimes changed prior to the advent of widespread, mechanized agriculture, long‐term records (i.e., centennial to millennial time scales) that capture past grassland fires before the industrial era can be analyzed. Such records come primarily from dendrochronological data based on fire‐scarred trees, or from paleoecological studies that employ charcoal accumulations in sediment. Such records are used to reconstruct different aspects of fire regimes—typically fire frequency over centennial scales in the case of fire‐scar data, and area or biomass burned and fire frequency over millennial scales in the case of charcoal records.

### 2.1 Multidecadal to centennial‐scale fire history records

Several key long‐term grassland burning analyses come from South African National Park records (van Wilgen et al. 2004; Archibald, Roy, Wilgen, Brian, & Scholes, 2009). Van Wilgen et al. (2004), for example, used a spatial database of all fires in the 2‐million ha Kruger National Park from 1957 to 2002 to examine the controls on fire regime under several different fire management approaches, including a lightning‐only regime. Regardless of approach, burned area was driven largely by variations in climate, particularly rainfall patterns that determined grass fuel levels. However, during the lightning regime (when prescribed burning was not conducted), a greater proportion of the park's area burned occurred during the dry season and thus suppression requirements increased. Regardless of management approach, fire‐return intervals were also largely determined by annual rainfall sequencing. The spatial heterogeneity and seasonal distribution of fires, however, was affected by management policies, and the current approach does include some prescribed burning to manage fuels (van Wilgen et al. 2004).

Some longer grassland fire history records have been obtained through dendrochronological methods. Fire scars on trees have long been used to reconstruct past fire events in a wide range of ecosystems, providing baseline estimates of fire‐return intervals for research and natural resource management (Abrams, 1992; Allen & Palmer, 2011; Guyette & Stambaugh, 2004; Ramankutty & Foley, 1999). In grasslands, where trees are necessarily limited, dendrochronology provides data on fire frequency, regeneration, and resilience in areas adjacent to grasslands or on their periphery (Arno & Gruell, 1983; Hély & Alleaume, 2006; Veblen, Kitzberger, Villalba, & Donnegan, 1999). As trees are scarce in grasslands by definition, tree‐based fire history reconstructions may over‐ or under estimate fire frequency in nearby or adjacent grasslands. Nonetheless, fire‐scar studies can serve as a rich source of information, particularly where millennial‐scale records from lakes, for example (hereafter paleofires), are scarce (Girardin et al., 2009; Swetnam, Allen, & Betancourt, 1999; Swetnam & Betancourt, 1998).

In central North America, extensive grasslands once stretched across 70 million hectares, but today less than 4% of the original Great Plains prairie remains (Murray et al., 2000). Lakes with continuous sediment records required for paleofire reconstructions are sparse here, particularly in drier areas where many lakes disappear during severe droughts. Analysis of fire scars from trees in one region has shown that a dramatic increase in fire frequency coupled with a decrease in fire intensity and/or size, and a change in the timing of fires, fundamentally altered the vegetation composition and structure (Allen & Palmer, 2011). Landholders in the Flint Hills of Kansas and Oklahoma, for example, now practice an annual, early‐spring burning which coupled with annual heavy grazing results in a relatively homogenous landscape as compared to pre‐industrial landscapes (Fuhlendorf & Engle, 2001). Fire occurrence here is not correlated with climate changes, such as pluvial or drought episodes, because a large majority of fires are now prescribed. Human impacts also dominate fire regimes in South Dakota, where fire‐scar records document a dramatic increase in burning near former grassland areas with the arrival of European settlers since the late 1800s (Brown & Sieg, 1999).

Patterns of increased burning by European settlers (e.g., to expand ranching), followed by decreased burning and subsequent tree invasion during the past century, have been documented in many fire‐scar studies and on multiple continents in addition to North America, including Patagonia (Alaback et al., 2003; Veblen & Lorenz, 1988), and Australia (Bowman, Walsh, & Prior, 2004; Bradstock, 2009). Similar to the fire history record from Kruger National Park, a modern climate‐fire connection is still evident in Asia. Fire‐scar data from Mongolian forests on the edge of grasslands, in particular, document a pattern of increased burning during dry years that followed wet years, despite extensive human activities (Gradel et al., 2017). Increased burning in dry years that follow wet years, in which biomass and thus abundant fuels have accumulated, are also recorded in fire‐scar studies from the southwestern United States (Swetnam & Betancourt, 1990), although in this case the data are from forests where grasses are an important component of the understory rather than from grasslands *per se*.

### 2.2 Millennial‐scale grassland fire records

Long fire history records from grasslands can provide insight into climatic and human impacts on grassland ecosystems (Aleman et al., 2013; Bhagwat, Nogue, & Willis, 2012; Carrión, Sánchez‐Gómez, Mota, Yll, & Chaín, 2003; Commerford, Leys, Mueller, & McLauchlan, 2016; Nelson et al., 2006; Umbanhowar et al., 2009) that extend beyond the typical age of trees, or that killed trees and thus prevented them from recording any signal. Multicentennial “megadroughts,” for example, or the effects of large temperature changes on fire and grasslands may be evident in sediment‐based paleofire reconstructions that will not be available or detectable in modern, historical, or tree‐ring data (Clark et al., 2002; Feurdean et al., 2013; Gavin et al., 2007; Grimm, Donovan, & Brown, 2011).

Millennial‐scale fire history reconstructions use variations in charcoal accumulation rates, or more simply, variations in charcoal abundances through time, to infer changes in biomass burning over thousands of years (Conedera et al., 2009; Lynch, Clark, & Stocks, 2004; Patterson, Edwards, & Maguire, 1987). Such Holocene paleofire records from grasslands often show a positive relationship between precipitation and biomass burning. As fires in grasslands are generally fuel limited, biomass burning tends to increase when conditions are relatively wet, which allows for a strong growing season and an increase in above‐ground biomass (Turner et al., 2008; Grimm et al., 2011). For instance, during the mid‐Holocene in the eastern Mediterranean, biomass burning was low in dry, fuel‐limited grassland sites, but subsequently increased with increasing precipitation that promoted the expansion of herb cover (Vannière et al., 2011). Likewise, at the grassland‐forest border in Minnesota, higher fuel loads and increased burning was associated with more favorable moisture conditions at ~4200BP, during the end of the mid‐Holocene warm period (Camill et al., 2003). An increase in the abundance of C4 grasses during this same time period is also documented and linked to greater moisture and high fire regime (Nelson, Hu, Tian, Stefanova, & Brown, 2004). Higher biomass during drier period of the experimentation in the Tallgrass Prairies of South Dakota, however, is associated with higher surface temperatures, and potentially with strong winds (Ohrtman, Clay, & Smart, 2015). Thus, past reconstructions suggest that climate–vegetation–fire interactions in grasslands are both spatially and temporally variable even without consideration of human impacts on fire regimes.

Fire history reconstructions have also been conducted on geological time scales. Charcoal in marine sediments, for example, has been analyzed to understand relationships between fire and the evolution of grasses, as well as climate–vegetation–fire interactions during periods of major Earth system changes. High charcoal abundances ca. 7–8 million years ago, for example, were coincident with the expansion of grasslands vegetation globally and suggest that fire has been a primary determinant of grassland distributions over millennia (Keeley & Rundel, 2005). The evolutionary history and fire associated with C3 and C4 species seems related to photosynthetic pathways, but as C4 andropogoneae grasses (a taxonomic tribe within the family Poaceae) are more flammable than most other clades (Ripley et al., 2015), they burn more extensively. On the other hand, in the tropics the C3/C4 balance is strongly linked to temperature gradients across elevations (Still et al., 2003), and so fire history does not appear to play a major role in their distributions here (Bremond, Boom, & Favier, 2012).

Charcoal abundances in a 170,000‐year marine sediment record off the western coast of Southern Africa suggest a strong role for climate in driving grassland distributions and fire cycles on orbital time scales. Here, charcoal elongation ratios, which are thought to reflect differences in material burned (i.e., periods with particles that are longer rather than wider are thought to reflect increased grass burning), oscillated on a 23,000‐year cycle corresponding to shifts in the Intertropical Convergence Zone that drove rainfall intensity and seasonality in the Orange River drainage basin. Changes in precession and their associated impacts on atmospheric circulation accounted for 57% of the variance in microcharcoal abundances here, presumably through their effects on vegetation and biomass productivity (Daniau et al., 2013). Phytolith and charcoal variations in marine sediments from the Amazon fan (Piperno, 1997) during the Last Glacial Maximum suggest that grassland fires were more frequent than during warmer periods, and suggest that changes in rainfall seasonality may also have been a dominant control on biomass burned in South America on orbital time scales.

## 3 GRASSLAND BIOMES AND FIRE HISTORY RECONSTRUCTIONS

### 3.1 Global charcoal database and grassland biomes

None of the many syntheses constructed from the Global Charcoal Database (e.g., Daniau et al., 2012; Marlon et al., 2016; Power et al., 2008; Vannière et al., 2016; Marlon et al., 2008) have focused on grassland systems. In part, this is due to the difficulties in identifying which sites should be considered “grassland” given that they can be defined in many ways and their spatial distributions vary through time. Some records that are defined today as grassland, for example, were forested in the past, and *vice versa*. Nonetheless, by limiting our focus to the Holocene, many sites that are considered grassland today have been grasslands throughout their history. In other cases, sites that may not be grassland today due to human influences are thought to be grassland for most of the Holocene prior to the Industrial Era. With these considerations in mind, we examine 121 sites from the GCDv3 (http://www.paleofire.org) that are considered to be modern‐day grasslands, or grasslands during the mid‐Holocene, when Northern Hemisphere growing season temperatures were generally warmer than today due to higher‐than‐present summer insolation. We examine fire history trends at these locations and discuss inferred fire–climate–vegetation–human dynamics in relation to the existing literature on grassland systems more broadly.

### 3.2 Classifying grassland biomes

Defining and identifying grasslands for analysis in a consistent fashion globally is a nontrivial challenge. Unlike forests, for which satellite‐based products such as the Global Land Cover dataset clearly defines eight distinct types (Bartholomé & Belward, 2005), there is a profusion of grassland definitions with no well‐established categorization system. In part, this is due to the difficulty in characterizing the limits of grasslands, which exist as a physiognomic continuum between forests and deserts (Dixon et al., 2014). Dixon et al. (2014) developed a broad definition of grassland as a distinct biotic and ecological unit, with similarities to savanna but distinct from woodland and wetland. While this definition seems suitable for comparing modern grassland ecosystems, past grassland distributions derived from pollen assemblages requires substantial and often complex interpretation.

Although pollen records can help define the dominant vegetation cover in forests and can also be used to define temporal transitions from forested to nonforested environments, no consensus exists on the dominance of grassland vegetation cover based on pollen assemblages, or the percentage of Non‐Arboreal Pollen (NAP) types. Even in grass‐dominated landscapes, pollen productivity is higher for woody taxa than herbaceous taxa due to differences in dispersal (i.e., by wind rather than insects) (Commerford, McLauchlan, & Sugita, 2013). In addition, NAP in vegetation history studies is typically considered to be part of the forest understory, or as the herbaceous vegetation around the depositional environment. As a result, it is very difficult to infer the landscape vegetation cover from NAP percentages alone. Moreover, grass pollen types differ substantially among regions, with the presence of emblematic species that compose grasslands in each region dependent on the continent and grassland formations (Lehmann et al., 2014; Oyarzabal, Paruelo, Federico, Oesterheld, & Lauenroth, 2008). Finally, pollen records are not evenly distributed across space, and thus, some regions of the world are lacking records from which vegetation changes through time can be inferred (Levavasseur, Vrac, Roche, & Paillard, 2012). Thus, although pollen data are our primary tool for reconstructing past vegetation dynamics, no reconstructions of forest versus grassland vegetation at the global scale are readily available for use in a global analysis of fire history during the Holocene.

Recognizing that no single scheme can perfectly define “grassland” sites, we chose four biome classification schemes that allow us to broadly distinguish between sites that can be considered modern and mid‐Holocene grasslands. We selected charcoal sites based on these classification schemes and developed composite fire history records for each scheme (Figure 1b) as follows:

1. The present grassland formation distribution follows the map published by Dixon et al. (2014) (hereafter D14).
2. The biome classifications from the contributors of the GCD generally represent the dominant vegetation for the site over the length of the record (hereafter bGCD).
3. The biome types modeled from climate and adjusted on the present‐day distribution of vegetation are drawn from Ramankutty and Foley (1999) (hereafter RF99).
4. The biome types modeled from climate and adjusted on the pollen records at 6000 years before present (hereafter year BP) are from Levavasseur et al. (2012) (hereafter L12).

These two latter classification schemes account for the possible under‐ or over‐representation of grassland biome distributions inferred from climate alone. Both the RF99 and L12 schemes avoid recent human impacts, such as agricultural areas, urban areas, and plantations.

The definition of grasslands as used in this manuscript is thus dependent on the classification analyzed (bGCD, D14, RF99, and L12). However, to be as consistent as possible within the four biome classifications, we choose biome types corresponding to the two same megabiomes for L12 and RF99: savanna and dry woodland, and grassland and dry shrubland; as defined by Harrison and Prentice (2003), thus, our four grassland categorizations include savannas and shrublands as well as unambiguously classified tropical and temperate grasslands (Table 1).

### 3.3 Analyses of charcoal signals in grassland biomes

The total charcoal quantities that accumulate in the sediments at a given location are controlled by many different factors, only some of which pertain to the local fire regime. Moreover, the controls on absolute charcoal quantities vary by region. In the western United States, for example, total charcoal abundances are determined by lake size, watershed size, local topography, and vegetation productivity (Marlon, Bartlein, & Whitlock, 2006). Elevation integrates many of these factors, and thus, sites at lower elevations often have more charcoal than those at higher elevations. In the eastern United States, topography is less important, and climate and fire‐regime characteristics exert a stronger influence on total charcoal (Clark & Royall, 1996). Different field, laboratory, and analytical approaches also influence absolute charcoal values in different studies. Thus, it is not possible to quantitatively reconstruct past fire‐regime changes based on charcoal abundances alone (Power, Marlon, Bartlein, & Harrison, 2010). It is still possible, however, to examine relative changes in charcoal accumulation rates and to interpret these as an indicator of changes in biomass burned, which have been calibrated to area burned in forested areas (Higuera, Whitlock, & Gage, 2011), and prairies (Leys, Brewer, McConaghy, Mueller, & McLauchlan, 2015; Leys, Commerford, & McLauchlan, 2017), over centennial and millennial time scales. To standardize these diverse records and make them comparable, we transformed the data with a Box‐Cox transformation and calculated Z‐scores with a base period from 12,000 to 250 cal year BP. The data were then smoothed using a 250‐year half window width. A bootstrap method was applied to assess the 95% confidence intervals around the mean. More details on the data transformation and the bootstrap can be found in Blarquez et al. (2014).

For each of the four classifications, we plotted the individual sites in a Hovmöller diagram (Hovmöller, 1949) to show the number of records spanning the past 12,000 years and to highlight the sites that contributed the most to each charcoal signal, that is, with the greater number of samples and/or the samples throughout the past 12,000 years. To show the differences in transformed charcoal values interpreted as biomass burned at each contributing site, we colored the individual samples from a site using their transformed values as follows: 0 ± 0.25 in gray, 0.5 ± 0.25 in light red, 1 ± 0.25 in dark red, −0.5 ± 0.25 in light blue, and 1 ± 0.25 in dark blue. The blue variations indicate samples that have below‐average biomass burned, while the red reflects values with above‐average biomass burned.

Charcoal trends for the four groups of grassland biomes based on L12, RF99, D14, and bGCD definitions and the forest biome (based on the L12 definition) were tested with a nonparametric Spearman test using the R package *pastecs* (R Core Team, 2014). A negative rho value, from −1 to 0, indicates declining charcoal values over time. In contrast, a positive rho value (from 0 to 1) indicates increasing charcoal values over time. A rho value close to 0 indicates no increase or decrease in the overall trend. *p*‐Values were calculated to assess the significance of the rho values. A change point analysis was used to assess significant changes over time, with no inference of trend direction (positive or negative) based on mean values using the R package *changepoint* (Grosjean & Ibanez, 2002; R Core Team, 2014). A penalty value was fixed at 0.05.

## 4 GLOBAL, FOREST, AND GRASSLAND FIRE HISTORIES

### 4.1 Grassland biome classifications

There are considerable differences in the labeling of the 121 grassland sites across the four different vegetation data classifications (Figure 1b). The biome descriptions are finer for the bGCD, with seven different biomes corresponding to nonforested lands, while biomes are coarser for RF99, which has three different nonforested biomes, and L12, which has only two different nonforested biomes (Table 1). The number of selected sites in the GCD is the smallest for bGCD biomes, and more than 2.5 times larger for the L12 biome scheme (Table 1). The number of sites per continent varies as does their temporal resolution (Supporting information Figures [Link], [Link], [Link], [Link]). Although the L12 biome maps encompass the largest number of sites, half of them are situated in dry woodlands in Australia, and the other four continents have similar numbers of sites when compared with the other biome maps. However, only about a third of “grassland” sites are shared between the four different sources (Figure 1b). The large differences in which sites are considered “grassland” can be explained by different factors. In all the classification schemes, ecotone sites may be included that are or were not grassland themselves, but are near or adjacent to grasslands. Such mismatches may be due to the coarseness of the classification scheme, mismatches that arise when overlaying a grid over point location data, or changes in time over vegetation. For example, it is well documented that Cygnet Lake in Yellowstone National Park, Wyoming in the United States has been surrounded by lodgepole pine forest throughout the Holocene (Millspaugh et al., 2000), but it appears in the L12 grassland biome because it was adjacent to extensive grasslands during the mid‐Holocene.

Other “grassland” sites that are known from detailed pollen analyses to be forested during the mid‐Holocene can very likely be identified in our classification schemes, and so the composite curves here should be considered to reflect a generalized rather than strictly defined representation of grassland burning over time. The D14 scheme reflects current (modern) grassland distributions. The RF99 scheme reflects a modeled distribution based on climate and corrected by the modern grassland distribution. Thus, neither D14 nor RF99 account for the past distribution of grasslands either by estimating biomes via pollen data as is done in the bGCD, or by inferring the potential distribution of grasslands through climate and pollen data analysis as in L12. A more careful and conservative representation of grassland sites would be quite useful but would require a large community effort that could provide site‐by‐site analysis and local expertise for the original records, which will prevail of reproducible analyses and comparisons. The L12 classification is arguably the best estimation to use for a global‐scale perspective on grassland burning during the Holocene because it does not rely on diverse and nonsystematic interpretations from GCD contributors and is not biased toward modern grasslands, even though the L12 classification includes dry woodlands, which results in more than 2.5 times the number of sites in Australia (Table 1) than the other classifications.

Regardless of the classification scheme applied, the number of GCD grassland sites is not proportional to the areal extent of grasslands on each continent or globally (in contrast to forested areas) based on their modern distribution (Dixon et al., 2014). More paleofire records exist in North America and Oceania regardless of the classification scheme, as compared with Eurasia, Africa, and South America (Table 1). The distribution of sites in Eurasia is also highly skewed, with most located in western Europe and Mongolia, and none in the temperate grasslands of Russia, Kazakhstan, or the Tibetan plateau (Figure 1b and Supporting information Figure S6). The absence of sites in these areas is not due to a lack of appropriate depositional environments (i.e., natural lakes, peats, bogs), but more likely results from their relatively remote locations. The African and South American continents are the least studied (Figure 4 and Supporting information Figure S5) because of limited local capacity for scientific research, political instabilities, lack of depositional environments, and protected areas for wildlife and habitats that prevent lake coring and the removal of sediments.

### 4.2 Millennial‐scale global trends

**Figure 2:**
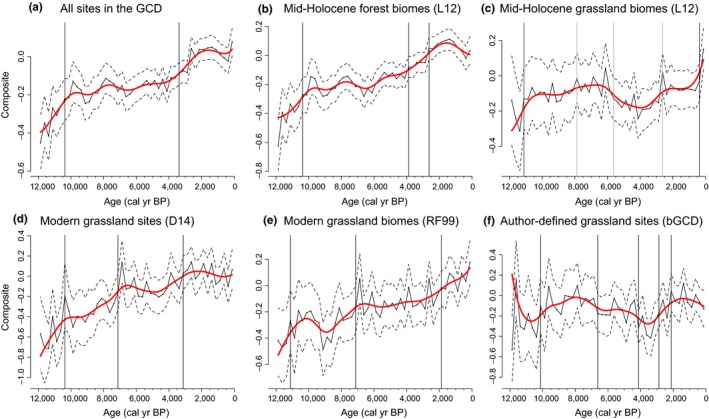
Normalized biomass burning trends for charcoal records using different classification schemes. (a) All sites in the Global Charcoal Database (GCD) version 3.0; (b**)** All sites within forested biomes following the Levavasseur et al. (2012) classification (L12); (c) All sites within nonforested biomes following the L12 classification; (d**)** All sites overlapping the current grassland distribution published by Dixon et al. (2014); (e**)** All sites within nonforested biomes following the Ramankutty and Foley (1999) classification (RF99); (f**)** All sites within nonforested biomes as described by contributors of the GCD (bGCD). Charcoal records are first rescaled to values between 0 and 1 (i.e., min‐max method), and then normalized using Box‐Cox followed by Z‐score transformations. The red lines represent the normalized charcoal data smoothed with a 250‐year half window, and the dashed lines represent the 95% confident intervals. The vertical solid lines correspond to significant change points with *p*‐values < 0.05 [Colour figure can be viewed at http://wileyonlinelibrary.com]

Charcoal trends show significant increases for all GCD sites, as well as for the forested sites following the L12 classification (rho of 0.90 and 0.89 respectively, Table 2). As proposed by Marlon et al. (2016), global biomass burning estimated from transformed charcoal data has increased over the past 12,000 years due to warming temperatures and human impacts. However, this trend is mostly represented by forested areas as shown in this study (Figure 2a and b). The grassland sites from the D14 and RF99 classifications also show an increase in charcoal (0.86 and 0.85, respectively, Table 2, Figure 2d and e). On the other hand, the grassland sites following the L12 and bGCD classifications show no significant increase or decrease for the 12,000‐year period (Figure 2c and f, Table 2). As discussed above, D14 and RF99 are both based on current distribution of grasslands, including cultivated lands for RF99 (Levavasseur et al., 2012). In the past decades, some forested lands have been converted to open areas such as cultivated lands (Murray et al., 2000; White et al., 2000). Conversely, some areas such as the European Alps or the Mediterranean show an opposite trend (Carcaillet & Muller, 2005; Genries, Muller, Mercier, Bircker, & Carcaillet, 2009; Leys & Carcaillet, 2016; Tinner, Ammann, & Germann, 1996). This change from a forested to more open landscape could have created an abrupt increase in the number of sites considered grassland areas today, but that were in fact forested landscapes in the recent past. In addition, the L12 classifications encompass all the shrublands and dry woodlands of the Australian continent, which leads to a unique charcoal trend during the two past millennia.

**Table 2:** Trends in transformed charcoal values over the past 12,000 years as determined by the application of a Spearman test, and associated *p*‐values. Negative values of rho, up to −1, indicate a decreasing trend, positive values of rho, up to +1, an increasing trend, and values around 0 indicate no positive or negative trend

Site selection	rho	*p*‐value
All sites of the GCD	0.89	≪0.001
Mid‐Holocene forest biomes (L12)	0.90	≪0.001
Mid‐Holocene grassland biomes (L12)	0.25	0.09
Modern distribution (D14)	0.86	≪0.001
Modern grassland biomes (RF99)	0.85	≪0.001
Author‐defined grassland sites (bGCD)	0.16	0.26

The two classifications that best represent grassland distributions in the past (L12 and bGCD) show no significant increasing trend over the past 12,000 years. Because global averages cannot, by definition, reveal regional heterogeneity, we examine the charcoal signals by continent. However, the strong differences in trends between the global forested versus grassland sites already highlight the value of analyzing data from forested and grassland landscapes separately, and also for treating these data separately in global syntheses, particularly in the context of reconstructing past carbon emissions. For example, while a global charcoal average can reveal the “net” effect of biomass burning—that is, whether a trend is positive or negative when everything is considered together at the planetary scale—if charcoal data were used to model CO_2_ emissions, biomass burning in forests versus grasslands sites would obviously need to be treated differently because grasslands emit much more carbon than forests when burned. Distinguishing the biomass burning trends in these two data types can thus support more nuanced global biomass burning and emissions reconstructions if used with models (e.g., van Marle et al., 2017). In light of this result, grassland records should be considered separately from forests in global‐scale analyses in general, although this may depend on the research question and time period of interest.

### 4.3 The three distinct periods in global grassland fire regimes

Change point analysis reveals periods of relatively rapid change in all of the global fire history reconstructions (Figure 2). A large increase in biomass burning at the beginning of the Holocene (between ca. 10 and 11,100‐year BP) is evident in the global reconstructions for both forested and grassland sites, with burning increasing slightly earlier in the grassland sites based on the L12 or RF99 classifications than in the D14 scheme or at the forested sites. After about 10,400 year BP for all GCD sites, all forest sites from L12, and all grassland sites from D14, and slightly after for the grassland sites from the bGCD, biomass burning becomes more stable. The increase in biomass burning at the onset of the Holocene is similar to the increase in forested sites and is consistent with warming climate conditions and changing broad‐scale precipitation patterns observed at longer time scales, especially drier conditions in the midlatitudes, and wetter conditions in some monsoon‐driven regions. In this hypothesis, grassland fires responded positively to warmer climate conditions broadly, and also to increases and decreases in precipitation depending on the region. In particular, because biomass burning is highest at intermediate levels of precipitation (Daniau et al., 2012) and the late glacial was generally dry, warmth and increasing moisture would have allowed more biomass growth in arid areas (Tierney & deMenocal, 2013) and thus more fire, and yet could have also increased fire in wetter areas as long as vegetation dried out seasonally. Burning could subsequently decline with continuing aridification, however, such as in the most arid phase of the mid‐Holocene in North America (Nelson, Verschuren, Urban, & Hu, 2012), or with the mixed effects of dry conditions and intense grazing in the same area (Umbanhowar et al., 2009).

The grassland curves from D14, L12, RF99, and bGCD showed two main changes in burning, with a decrease in charcoal Z‐scores after the early Holocene, the first between 8000‐ and 7000‐year BP, and the second between 4000‐ and 3000‐year BP. The global change in grassland fire dynamics around 8000‐ to 7000‐year BP is not shared with the forested sites from L12, or with all GCD sites combined. In the Holocene, several shifts from grassland to forest have been reported in different parts of the world, corresponding mostly to changes in climatic conditions. The maximum extent of grassland cover in the United States is about 8000‐year BP, corresponding to the beginning of the warmest and driest period in North America in the Holocene (Shuman & Marsicek, 2016). At this time, grasslands expanded as did fire, consistent with previous fire history reconstructions (Nelson et al., 2006; Walsh et al., 2008; Power et al., 2011).

The charcoal signal is stable for both L12 and bGCD grassland sites during the mid‐Holocene, and is likely linked to drier climatic conditions especially in northern temperate zones that led to lower fuel availability and burning in grassland areas (Brown et al., 2005a; Clark et al., 2002; Nelson et al., 2012), coupled with increased burning in forested sites. Increased biomass burning in D14 and RF99 sites in particular include sites comprised of forest at that time, but that have been converted to grasslands today. As an example, several long fire history records have been constructed from grassland and steppe vegetation in the loess plateau of China that point to climate changes as a driver of vegetation and fire‐regime changes. Wang and Li (2007) found that the rates of black carbon mass sedimentation rates (BCMSR) were 2–3 times higher in cold, dry glacial conditions, for example, than in interglacial periods. However, the relative importance of temperature versus precipitation in driving such patterns remains unclear, as is the relative extent of steppe versus grassland vegetation in the past.

### 4.4 Biomass burning from mid‐Holocene grassland biomes (L12) by continent

#### 4.4.1 Oceania (Australia)

**Figure 3:**
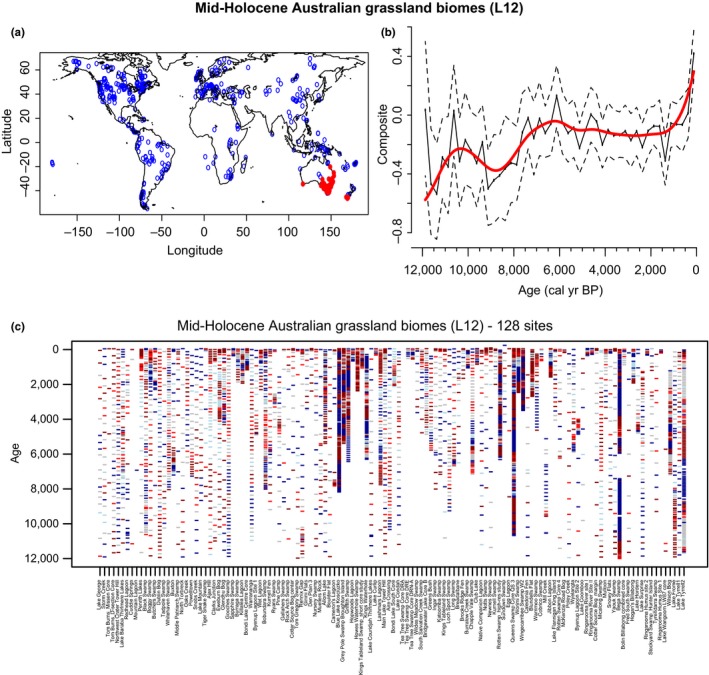
Summary of the charcoal signal of grassland sites following the Levavasseur et al. (2012) classification (L12) in Oceania (Australia). (a) Distribution map of the selected sites in red, compare to all the sites in the GCD in blue. (b**)** Normalized charcoal signal of the selected charcoal records on the last 12,000 years. Charcoal records have been standardized (min max method), and normalized (Box‐Cox and Z‐score transformations). The red lines represent the normalized charcoal data smoothed at 250‐year windows, and the dashed lines represent the 95% confident intervals. (c**)** Hovmöller‐type diagram with Z‐scores of transformed charcoal records from the 128‐selected series. Tick marks represent individual samples with colors underlining periods with dominant positive (red) or negative (blue) Z‐score values [Colour figure can be viewed at http://wileyonlinelibrary.com]

The Oceanian grassland sites are the most numerous, representing 48% of the charcoal records from the L12 grassland biomes (Table 1), all situated in Australia (Figure 1b). As a result, the reconstructions from the global L12 grassland sites and the Australian sites are highly similar (Figures 2c and 3). There is no significant trend for the full period, but there is a change point at ca 400‐year BP that marks a large increase in biomass burning. A previous synthesis of fire regimes in Australasia (Mooney et al., 2011) based on 223 charcoal records from the GCD version 2 examined biomass burning in the context of climate and anthropological data. The authors conclude that overall the fire regimes in Australasia based on the last 70,000 years predominantly reflect climate variability, with no distinct change at the arrival of human at 50,000‐year BP, although human activities could have nonetheless played important local roles in determining fire regimes. However, a recent increase in biomass burning beginning around 200‐year BP is interpreted as an unambiguous signal of human influence, whether directly through vegetation and land‐use changes or indirectly through anthropogenic climate changes that affect fire‐regime characteristics. In the past century, higher precipitation in preceding years has promoted fire events during warmer months in this region (Edwards et al., 2008; Greenville, Dickman, Wardle, & Letnic, 2009), but an increase in fire frequency during the cooler months has also been reported due to management approaches in central Australia (Edwards et al., 2008).

#### 4.4.2 South America

**Figure 4:**
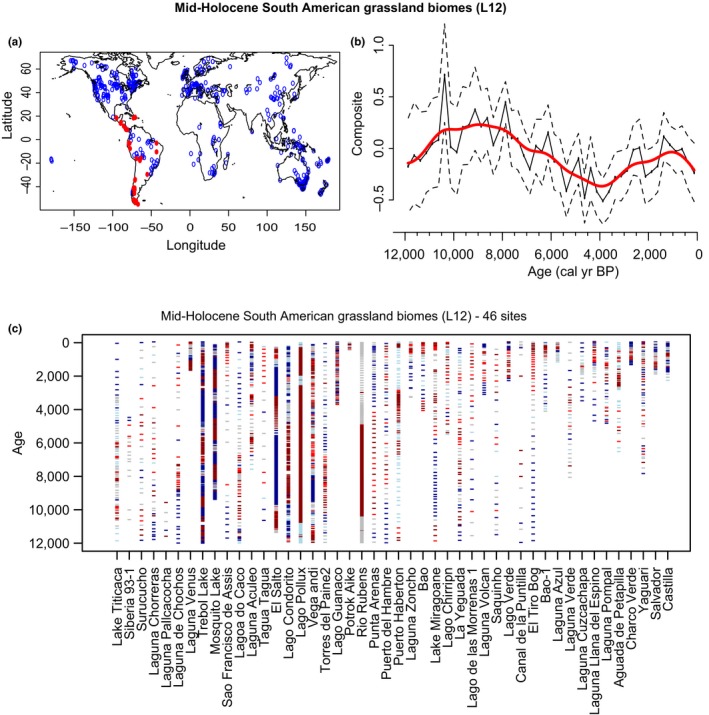
Summary of the charcoal signal of grassland sites following Levavasseur et al. (2012) classification (L12) in South America. (a**)** Distribution map of the selected sites in red, compare to all sites in the GCD in blue. (b**)** Normalized charcoal signal of the selected charcoal records on the last 12,000 years. Charcoal records have been standardized (min max method), and normalized (Box‐Cox and Z‐score transformations). The red lines represent the normalized charcoal data smoothed at 250‐year windows, and the dashed lines represent the 95% confident intervals. (c**)** Hovmöller‐type diagram with Z‐scores of transformed charcoal records from the 48‐selected series. Tick marks represent individual samples with colors underlining periods with dominant positive (red) or negative (blue) Z‐score values [Colour figure can be viewed at http://wileyonlinelibrary.com]

In South America, the charcoal trend is very different trend from the global L12 and the Australian continent trend (Figure 4). There is an increase in burning from 12,000 to 10,000 year BP likely linked to the warmer climatic conditions, longer growing season, increased biomass productivity, and thus fuel availability at the onset of the Holocene compared to the late glacial (Power et al., 2008). However, the decrease in charcoal after 10,000‐year BP may reflect another driver overriding temperature, such as precipitation reductions that reduce fuel availability, or changes in human activities (Moreno et al., 2000; Whitlock et al., 2007). Analysis of diverse paleoenvironmental data from multiple sites in Patagonia, for example, demonstrates that fuel discontinuity played a greater role in driving biomass burning trends here than other factors such as climate variability or human activities (Iglesias and Whitlock, 2014). Reductions in moisture availability were reported from 10,000‐ to 6000‐year BP in Brazil (Cruz et al., 2005) and may have decreased fuel availability in grassland areas enough to limit fire, for example. Wetter conditions from 4,000‐year BP to present are correlated with higher fuel loads and biomass burning in southern South America (Iglesias et al., 2014). South American fire frequencies over the Holocene are sensitive to the alternation of dry and wet conditions, although human activities may have influenced fire locally since the onset of the Holocene (Behling, Pillar, & Bauermann, 2005). Human impacts may also have reinforced climate influences on grassland fires on this continent (and elsewhere), with increased fire ignitions by humans during wetter periods (Behling et al., 2005).

#### 4.4.3 North America

**Figure 5:**
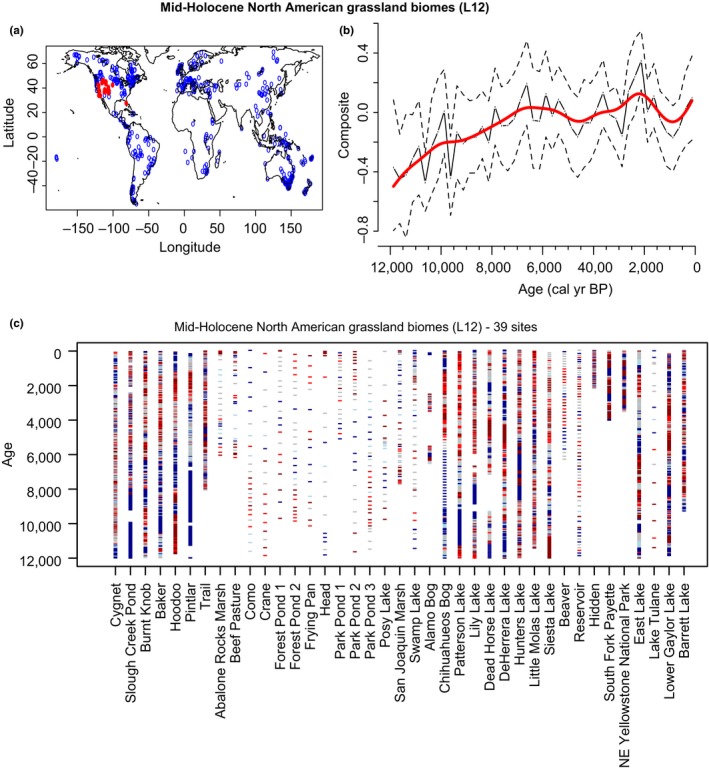
Summary of the charcoal signal of grassland sites following Levavasseur et al. (2012) classification (L12) in North America. (a**)** Distribution map of the selected sites in red, compare to all the sites in the GCD in blue. (b**)** Normalized charcoal signal of the selected charcoal records on the last 12,000 years. Charcoal records have been standardized (min max method), and normalized (Box‐Cox and Z‐score transformations). The red lines represent the normalized charcoal data smoothed at 250‐year windows, and the dashed lines represent the 95% confident intervals. (c**)** Hovmöller‐type diagram with Z‐scores of transformed charcoal records from the 39 selected series. Tick marks represent individual samples with colors underlining periods with dominant positive (red) or negative (blue) Z‐score values [Colour figure can be viewed at http://wileyonlinelibrary.com]

Biomass burning is increasing during the Holocene for most areas in North America (Blarquez et al., 2015; Marlon et al., 2013), compared to biomass burning in grassland biomes from L12 only, which shows a smaller increase (Figure 5) including a period of stable fire activity from 6,000‐ to 3,000‐year BP. Regional coherency in past fire occurrence has been reported in the northwestern United States, likely driven by regional climate changes (Marlon et al., 2006; Whitlock et al., 2008). High fire activity in present‐day summer‐dry areas show a period of protracted high fire activity during the early Holocene that was attributed to intensified summer drought in the western summer‐dry region (Brunelle et al., 2005). Relatively high biomass burning during the middle Holocene in western North America is supported by evidence for frequent fires in grasslands and savanna (Walsh et al., 2008; Power et al., 2011).

Higher fire activity in the late Holocene is observed in many regions in North America, but peak burning is earlier in the interior central region than in more coastal areas (Marlon et al., 2013). Maximum burning around 2000 years ago in the northwestern United States has been observed in both forest and grassland biomes, and has been attributed in some cases to climate changes such as the Roman Warm Period (Hallet et al., 2003; Lepofsky et al., 2005) and the Medieval Climate Anomaly associated with warm, dry conditions about 1000 years ago (Whitlock et al., 2008), and in other cases to Native American fire use, particularly in (forested) coastal regions (Brown and Hebda, 2002) but also in grassland river valleys (Walsh et al., 2010) and inland areas (Roos et al., 2010; Scharf, 2010; Walsh et al., 2015).

#### 4.4.4 Eurasia and Africa

Given the number of sites available in grassland biomes in Eurasia (26) and in Africa (23), and their temporal resolutions, interpretations have to be made with caution (Supporting information Figures S5, S6). The Eurasian sites are represented mostly by Mongolian sites, primarily in the western mountainous region (Supporting information Figure S6). Eighteen of the 26 sites record less than 8,000 years of history, including six sites recording only the past 2,000 years. The increase in biomass burning from 12,000 to 8,000 years, commonly explained by the maximum summer insolation and warmer conditions at the onset of the Holocene (Vannière et al., 2011), is driven by Sicilian sites, Arabic sites, and Asian sites on isolated islands. During the past 8,000 years, biomass burning remains stable, but is comprised of Mongolian sites recording very few charcoal particles (Umbanhowar et al., 2009), due to the high intensity of pasture activities that reduce available fuels.

More sites in Africa record the past 12,000 years of fire history, but the temporal and spatial resolution, as well as the number of samples per site is very low. Many variations in the fire activity could have therefore been missed, especially between 8,000 and 4,000 years before present, where biomass burning appears lower but where data are particularly sparse (Supporting information Figure S5).

## 5 FUTURE RESEARCH DIRECTIONS

### 5.1 Interpretation of charcoal as a fire proxy in grassland systems

#### 5.1.1 More charcoal data

The registration of fire‐regime changes in lake sediments from grassland ecosystems remains poorly understood. A key fire research priority is thus to establish working definitions of grassland ecosystems as distinct from forest ecosystems appropriate for long‐term (paleo) studies. The reconstruction of fire history and the distinction of fuels burned, from charcoal trends and morphology respectively (see Discussion, below), can be useful for defining grassland and forest landscapes through time. Through those two charcoal‐based metrics, insights into potential impacts of human activities on fuel burned and the importance of fire in opening forested landscapes or maintaining grasslands might be better understood. There is also a critical need to develop more paleofire records in modern and Holocene grassland systems, especially in the tropics and in Asia, but even on continents that generally have better coverage, such as in North America and Europe as the charcoal records in these two continents are mostly in forested environments or at the edge of grasslands. Our results also point to important differences in grassland distributions given different biome classifications, and emphasize the variability in these classification systems depending on the time period of interest. In order to better understand these systems and their dynamics, classification systems must be chosen carefully, particularly if modern‐day grassland systems are being considered, which often do not reflect natural long‐term processes at work and do not correspond to the concept of old‐growth grasslands (Veldman et al. 2016).

#### 5.1.2 Calibration of charcoal data to better understand grassland fire regimes

There are many charcoal records that already exist from grasslands, but the interpretation of such records is difficult due to our limited understanding of how burned material is incorporated into sediments during and after grassland fires. Calibration studies that examine how charcoal particles from recent fires are produced, transported, deposited, and incorporated into sediments (i.e., “taphonomy”) in grassland versus forested ecosystems would greatly improve our ability to accurately reconstruct grassland fire history (Leys et al., 2015, 2017).

Paleofire reconstructions require a robust method for analyzing high‐resolution charcoal series in lake or peat sediments (Whitlock & Anderson, 2003). Charcoal counts and variability depend in part on fire intensity and burned area, as well as on the associated vegetation that determines fuel structure, distribution, and quality (Higuera, Sprugel, & Brubaker, 2005; Pitkanen, Lehtonen, & Huttunen, 1999). Therefore, charcoal amounts alone do not reflect fire history *per se*; as these values can vary over time and be biased by environmental parameters acting on charcoal taphonomy. In forested areas that experience stand‐replacing fires for example, high‐resolution charcoal series can be decomposed into peak and background components, with peaks of charcoal used as indicators of discrete fire events or episodes (i.e., multiple large fires within a year or few years) (Higuera et al., 2011). However, grassland ecosystems that burn annually or more often than can be distinguished given the sediment sampling resolution may result in high values of charcoal “background” levels in lake sediments. In these cases, it is difficult to know what a “peak” in charcoal actually reflects, especially if the sedimentation rate is slow, meaning that many fires are reflected in each sample. Such conditions make it difficult, if not impossible, to detect individual fire events or even episodes of high fire frequency (Brown et al., 2005b; Clark, Grimm, Lynch, & Mueller, 2001; Commerford et al., 2016; Nelson et al., 2012). Fire reconstructions in grassland areas are thus mainly based on charcoal signal variations, and the interpretations are as diverse as the uses of different terms, such as “fire importance” and “fire activity” (e.g., Brown et al., 2005b; Clark et al., 2001; Nelson et al., 2006) or even “fire frequency” (e.g., Behling, Pillar, Muller, & Overbeck, 2007; Chang Huang et al., 2006). A consistent pattern of interpretation that has emerged from many grassland fire history studies, however, is that charcoal abundances reflect changes in the quantity of biomass burned (which may or may not reflect changes in *area* burned) over multidecadal to multimillennial scales (e.g., Camill et al., 2003; Umbanhowar, 2004; Brown et al., 2005a,b; Umbanhowar et al., 2009; Power et al., 2011; Daniau et al., 2012; Colombaroli, Ssemmanda, Gelorini, & Verschuren, 2014; Aleman et al., 2013; Leys & Carcaillet, 2016; Leys et al., 2017).

#### 5.1.3 New perspectives with charcoal morphotypes

A few published records from sites that are not in grasslands but that have a grass component have provided separate measurements of herbaceous versus woody charcoal based on visual observation of morphology. These measurements can provide information about changes in dominant fuel sources over time, offering insights into fire‐regime characteristics such as fire intensity or burn severity. For instance, postglacial charcoal and pollen data from Lago el Trébol in Patagonia, a midelevation forested site (Whitlock et al., 2006) on the Argentine side of the Andes, show that background charcoal was generally lowest when the proportion of grass to woody charcoal was the highest, perhaps indicating shifts in fire types from crown fires to understory fires, indicating a decrease in fire severity. Colombaroli et al. (2014) reconstructed the fire history of two sites in wet and dry savanna in Uganda and Kenya, and show that a doubling of mean charcoal accumulation rates occurred during a period when >80% of the charcoal was derived from grasses, countering a common misconception that grass burning does not produce much charcoal. A charcoal calibration study from Kruger National Park, South Africa, further found that the abundance of macrocharcoal particles reflected variations in fire intensity more strongly it reflected fire proximity or burned area (Duffin, Gillson, & Willis, 2008).

Although charcoal morphotypes have been analyzed primarily in forested environments, a recent study suggests they may be inaccurate in grass‐dominated landscapes (Leys et al., 2015). Some studies have shown that the elongation of charcoal particles, however, or the width to length ratio, is an accurate proxy to reconstruct fuel sources, either woody or herbaceous (Aleman et al., 2013; Leys et al., 2017; Umbanhowar & Mcgrath, 1998). In New Zealand, monosaccharide anhydrides, including levoglucosan, mannosan, and galactosan, are used as molecular markers of biomass burning in sediment cores. Levoglucosan/mannosan and levoglucosan/(mannosan + galactosan) ratios in particular allowed the reconstruction of grassland versus hardwood fuel sources, which compared well with variations in macroscopic charcoal abundances (McWethy et al., 2009; Kirchgeorg, 2015). Vanillic acid and other fire proxies found in ice cores are increasingly being used to reconstruct long‐term variations in biomass burning (Grieman, Aydin, Isaksson, Schwikowski, & Saltzman, 2018; Grieman, Aydin, McConnell, & Saltzman, 2018). Some of these proxies are only produced by particular types of vegetation, which may help us better disentangle grassland fire history from charcoal data in the future. Vanillic acid, for example, is mainly produced by the incomplete combustion of conifers. In contrast, p‐HBA is a primary indicator for grass burning (Simoneit, 2002). Thus, future comparisons of GCD data with ice‐core fire proxies may yield new insights into both local and broad‐scale trends in paleofire history.

### 5.2 Pollen/charcoal integration to better define grassland landscapes

The grassland definition of Dixon et al. (2014) is suitable for comparison with modern grassland ecosystems, but needs to be developed for past grasslands, as has begun with pollen assemblages using L12 modeled biomes (Levavasseur et al., 2012). Nonetheless, the authors acknowledged that the model predicted some points in northeastern Europe and Spain as temperate or boreal forests instead of temperate forests or grasslands and dry shrublands, possibly due to difficulties in accounting for human impacts, and the absence of consideration of soil proprieties in the model.

Many studies designed to calibrate pollen assemblages to vegetation abundance and landscape structure (Commerford et al., 2013; Sugita, 1993, 1994; Tauber, 1974) have been conducted in forests. Such studies need to be extended to grassland environments, by comparison of subsurface pollen assemblages with present‐day grassland ecosystems as defined by Dixon et al. (2014). If we can assume that this relationship is constant through time, then we can apply the scaling from the modern day to the past, and at least reconstruct changes in forest versus grassland vegetation in the paleo record (Williams & Shuman, 2008). Recent studies aimed to address this challenge by calibrating the herbaceous pollen taxa from depositional environment with the surrounding vegetation (Commerford et al., 2013; McLauchlan, Commerford, & Morris, 2013) and demonstrated clear correlations between pollen taxa variations and vegetation assemblages. Another possibility is to establish the proportion of tree to nontree pollen in surface samples, and then calibrate this against satellite‐based estimates of tree cover (e.g., Tarasov et al., 2007). This ratio can be used to account for the over‐representation of trees in pollen spectra due to their high pollen productivity compared with grasses/forbs. Because herbaceous species are very sensitive to climate variations, there is also an opportunity to link pollen assemblages with climatic conditions, allowing precipitation reconstructions in these areas (Commerford et al., 2017).

### 5.3 Other controls of fire regimes in grassland systems: herbivores

Fire intensity (the rate of energy released along a fire front) and frequency in grasslands today is often fuel dependent (Keeley & Rundel, 2005; Whelan, 1995), and fuel loads are tied to net primary productivity (NPP), which is both precipitation and grazing dependent (Briggs et al., 1997; Christensen, Coughenour, Ellis, & Chen, 2004; Knapp, Briggs, Hartnett, & Collins, 1998; Scurlock & Hall, 1998; Shinoda, Ito, Nachinshonhor, & Erdenetsetseg, 2007).

It has already been demonstrated that bison, for example, today play an important role in grassland productivity and on fuel availability (e.g., Leys et al., 2015). Fire exclusion or removal of herbivores can result in rapid transitions from grassland to forest, with associated losses of herbaceous plant diversity (Scasta et al., 2016; Veldman et al., 2015). Overgrazing and increases in atmospheric CO_2_ can also lead to forest encroachment, despite frequent fires (Brunelle, Minckley, & Delgadillo, 2015). In yet other systems, increasing grazing densities, while reducing fire, may increase grasslands (Archibald, 2008). The combined effect of herbivory and fire on grasslands is thus far from predictable and remains unclear. New proxies and a broader interpretation of existing proxies will be required for a better understanding of the impacts of grazing on fire and vegetation (Craine & McLauchlan, 2004).

A recent proxy for past grazing intensity is dung fungal spores from herbivores, which can be identified and counted on pollen slides (Etienne, Wilhelm, Sabatier, Reyss, & Arnaud, 2013; Gill et al., 2013; Raper & Bush, 2009). Dung fungal spores were used recently as a proxy to show that the density of grazers is clearly not independent of climate‐driven changes in NPP (Davis & Shafer, 2006). Ancient sedimentary DNA has also been used to reconstruct the fauna distribution and evolution since the Late Pleistocene (Willerslev & Cooper, 2005). This technique can also be applied to sedimentary sequences to assess the diversity of the fauna, including herbivore megafauna. Finally, in New Zealand, a novel approach was recently used to compare the fire history with human presence and grazing activity identified with Fecal sterols in a sediment record (Kirchgeorg, 2015).

### 5.4 Prescribed burning versus natural wildfires

Grasslands have been managed using fire for millennia, and prescribed burning remains an integral part of grassland maintenance today, especially for limiting the rapid spread of uncontrolled wildfires. Prescribed burns in any vegetation type are typically conducted only under very specific and highly stable meteorological conditions, usually during the growing season when there is no wind, humidity is not too low, and the vegetation is moist (Fernandes & Botelho, 2003; Valkó, Török, Deák, & Tóthmérész, 2014). In European grasslands, for example, burning is usually organized by plots, in which all the above‐ground biomass is burned, resulting in relatively homogeneous fire impacts. Because external factors such as wind speed and vegetation moisture are highly controlled, fire spread is closely managed, and fire intensity is largely consistent in a given area (Pastro, Dickman, & Letnic, 2011; Valkó et al., 2014). Natural fires in uncontrolled conditions in contrast generally have higher severity, and can prevent stand establishment. On the other hand, the volatilization of nutrients such as Nitrogen is more likely in more intense fires (DeBano, 1991; Neary, Klopatek, DeBano, & Ffolliott, 1999), and soils can be consumed during combustion, releasing more carbon in the past than today. Pellegrini et al. (2018) shows that the time of fire treatment is impacted more the grassland, savannas, and broadleaf forests than the neddleleaf forests on the Carbon, Nitrogen and Phosphorous storage in soils. Paleofire impacts on vegetation, carbon, and other nutrients in the systems are therefore likely to be different than those observed and monitored today (Dijkstra, Wrage, Hobbie, & Reich, 2006; Hernández & Hobbie, 2008; Ohrtman et al., 2015; Reich, Peterson, Wedin, & Wrage, 2001), which underscores the need to improve our understanding of grassland fire history and the potential consequences of future human impacts and climate change.

### 5.5 Paleofire simulations in earth system models

Fire is commonly understood to be the primary pathway of carbon release in grasslands. The rapid regrowth of vegetation after fires in savanna grasslands, however, is assumed to sequester an equivalent amount of atmospheric CO_2_ to what was released during burning, resulting in no net change to the CO_2_ budget in the absence of abrupt climate changes or land‐use changes (Harrison & Bartlein, 2012). Thus, grassland systems that have been established for millennia and have experienced high fire frequencies constitute an important carbon reserve, storing up to 30% of global soil carbon (Figure 3, Scurlock & Hall, 1998; Murray et al., 2000; Veldman et al., 2015). The carbon stock in grassland areas is thus 80% in the roots, and in the organic matter of the soil (Murray et al., 2000; Scholes & Hall, 1996). The conversion of grassland to cropland and pasture has a primary consequence of changing carbon‐rich reservoirs to systems that stock less carbon per unit area of land. Today, the estimation of the grassland contribution to the carbon sink remains unknown, but some estimations based on a missing part of the carbon sink suggest that it may be as much as 0.5 Gt per year (Scurlock & Hall, 1998). The large amount of land area covered by grasslands as well as the relatively unexplored potential for grassland soils to store carbon has increased interest in the carbon cycles of these ecosystems.

While global fire modeling has advanced rapidly in the past decade (Rabin et al., 2017), modeling global grassland burning in particular is a new frontier (Lasslop et al. 2016), particularly on long time scales. As a result, some assumptions about global biomass burning that are based on our understanding of forest fires may need to be revised as models develop. For example, while the likelihood of fire increases since the last burn in forests, this is not true in grasslands, where the opposite relationship is more likely (Bond, Woodward, & Midgley, 2005). Improving reconstructions of paleofires in grasslands will improve our ability to examine their role in the carbon cycle and will also facilitate examination of how fire in grasslands interacts with climate and vegetation changes, currently being explored in Earth system models (e.g., Lasslop et al. 2016).

## 6 CONCLUSION

Relatively few fire history reconstructions and calibration studies have been conducted in grassland as compared with forest ecosystems to date. As a result, many assumptions have been made about grassland fires based on limited evidence, or on evidence more appropriate to woodlands and forests. Our synthesis represents a first attempt to consider grassland burning independent of forest burning globally and suggests that the history of biomass burning in these two biome types was significantly different from one another. Understanding why, and which factors were most important in determining the different trajectories of burning, will require both more paleodata as well as more modern calibration data to improve our ability to interpret paleofire records from grasslands. Each grassland ecosystem is different, but all are in decline today due to the expansion of forests and croplands, which store less carbon in soil, provide fewer habitats for fauna, and contain less flora diversity. Advancing our knowledge of grassland fire history would allow us to address questions about the role of fire, carbon emissions and storage, and the role of herbivores in grassland systems more effectively.

Given our current understanding of Holocene grassland fire history based on the four biome classifications presented here, trends in grassland burning differed from those in forested areas throughout the Holocene, but particularly in the mid‐ and late Holocene. In light of this result, we encourage grassland records to be considered separately from forest systems in global‐scale analyses in general, although this depends on the research question and the time period of interest. Further work on charcoal calibration and comparison with modern fire data, in conjunction with additional analyses of charcoal morphologies are important areas for future paleofire research. Coupling paleofire data with modeling efforts, both regionally and at the global scale, are also needed in order to better understand the carbon cycle in grasslands and interactions between fire, vegetation, biodiversity, and climate change more broadly.

## CONFLICT OF INTEREST

None declared.

## AUTHOR CONTRIBUTIONS

B.A.L., J.R.M., and B.V. designed the research, B.A.L., J.R.M., B.V., and C.U. performed research and provide data, B.A.L. and B.V. contributed analytical tools; B.A.L analyzed data; and B.A.L., J.R.M., B.V., and C.U. wrote the study.

## DATA ACCESSIBILITY

Site location, and charcoal data for all the sites presented in this study: Global Charcoal Database website: http://www.paleofire.org.

## References

[ece34394-bib-0001] Abrams, M. D. (1992). Fire and the development of oak forests. BioScience, 42, 346–353. 10.2307/1311781

[ece34394-bib-0002] Alaback, P. , Veblen, T. T. , Whitlock, C. , Lara, A. , Kitzberger, T. , & Villalba, R. (2003). Climatic and human influences on fire regimes in temperate forest ecosystems in North and South America In Bradshaw Gay A. & Marquet Pablo A. (Eds.), How landscapes change (pp. 49–87). Berlin: Springer 10.1007/978-3-662-05238-9

[ece34394-bib-0003] Aleman, J. C. , Blarquez, O. , Bentaleb, I. , Bonté, P. , Brossier, B. , Carcaillet, C. , … Lefèvre, I. (2013). Tracking land‐cover changes with sedimentary charcoal in the Afrotropics. Holocene, 23, 1853–1862. 10.1177/0959683613508159

[ece34394-bib-0004] Allen, M. S. , & Palmer, M. W. (2011). Fire history of a prairie/forest boundary: More than 250 years of frequent fire in a North American tallgrass prairie. Journal of Vegetation Science, 22, 436–444. 10.1111/j.1654-1103.2011.01278.x

[ece34394-bib-0005] Archibald, S. (2008). African grazing lawns—How fire, rainfall, and grazer numbers interact to affect grass community states. Journal of Wildlife Management, 72, 492–501. 10.2193/2007-045

[ece34394-bib-0006] Archibald, S. , Roy, D. P. , Wilgen, V. , Brian, W. , & Scholes, R. J. (2009). What limits fire? An examination of drivers of burnt area in Southern Africa. Global Change Biology, 15(3), 613–630. 10.1111/j.1365-2486.2008.01754.x

[ece34394-bib-0007] Arno, S. F. , & Gruell, G. E. (1983). Fire history at the forest‐grassland ecotone in southwestern Montana. Rangeland Ecology & Management/Journal of Range Management Archives, 36(3), 332–336.

[ece34394-bib-0008] Balch, J. K. , Bradley, B. A. , D'antonio, C. M. , & Gómez‐Dans, J. (2013). Introduced annual grass increases regional fire activity across the arid western USA (1980–2009). Global Change Biology, 19, 173–183. 10.1111/gcb.12046 23504729

[ece34394-bib-0009] Bartholomé, E. , & Belward, A. S. (2005). GLC2000: A new approach to global land cover mapping from Earth observation data. International Journal of Remote Sensing, 26, 1959–1977. 10.1080/01431160412331291297

[ece34394-bib-0010] Behling, H. , Pillar, V. D. , & Bauermann, S. G. (2005). Late Quaternary grassland (Campos), gallery forest, fire and climate dynamics, studied by pollen, charcoal and multivariate analysis of the São Francisco de Assis core in western Rio Grande do Sul (southern Brazil). Review of Palaeobotany and Palynology, 133, 235–248. 10.1016/j.revpalbo.2004.10.004

[ece34394-bib-0011] Behling, H. , Pillar, V. D. , Muller, S. C. , & Overbeck, G. E. (2007). Late‐Holocene fire history in a forest‐grassland mosaic in southern Brasil: Implications for conservation. Applied Vegetation Science, 10, 81–90. 10.1111/j.1654-109X.2007.tb00506.x

[ece34394-bib-0012] Bhagwat, S. A. , Nogue, S. , & Willis, K. J. (2012). Resilience of an ancient tropical forest landscape to 7500 years of environmental change. Biological Conservation, 153, 108–117. 10.1016/j.biocon.2012.05.002

[ece34394-bib-0013] Blarquez, O. , Ali, A. A. , Girardin, M. P. , Grondin, P. , Fréchette, B. , Bergeron, Y. , & Hély, C. (2015). Regional paleofire regimes affected by non‐uniform climate, vegetation and human drivers. Scientific Reports, 5, 13356 10.1038/srep13356 26330162 PMC4557068

[ece34394-bib-0014] Blarquez, O. , Vannière, B. , Marlon, J. R. , Daniau, A.‐L. , Power, M. J. , Brewer, S. , & Bartlein, P. J. (2014). paleofire: An R package to analyse sedimentary charcoal records from the Global Charcoal Database to reconstruct past biomass burning. Computers & Geosciences, 72, 255–261. 10.1016/j.cageo.2014.07.020

[ece34394-bib-0015] Bond, W. J. (2008). What limits trees in C 4 grasslands and savannas? Annual Review of Ecology Evolution and Systematics, 39, 641–659. 10.1146/annurev.ecolsys.39.110707.173411

[ece34394-bib-0016] Bond, W. J. , & Keeley, J. E. (2005). Fire as a global “herbivore”: The ecology and evolution of flammable ecosystems. Trends in Ecology & Evolution, 20, 387–394. 10.1016/j.tree.2005.04.025 16701401

[ece34394-bib-0017] Bond, W. J. , Woodward, F. I. , & Midgley, G. F. (2005). The global distribution of ecosystems in a world without fire. New Phytologist, 165(2), 525–538. 15720663 10.1111/j.1469-8137.2004.01252.x

[ece34394-bib-0018] Bowman, D. M. J. S. , Walsh, A. , & Prior, L. D. (2004). Landscape analysis of Aboriginal fire management in Central Arnhem Land, north Australia. Journal of Biogeography, 31, 207–223. 10.1046/j.0305-0270.2003.00997.x

[ece34394-bib-0019] Bradstock, R. A. (2009). Effects of large fires on biodiversity in south‐eastern Australia: Disaster or template for diversity? International Journal of Wildland Fire, 17, 809–822.

[ece34394-bib-0020] Bremond, L. , Boom, A. , & Favier, C. (2012). Neotropical C3/C4 grass distributions–present, past and future. Global Change Biology, 18, 2324–2334. 10.1111/j.1365-2486.2012.02690.x

[ece34394-bib-0021] Briggs, J. M. , Rieck, D. R. , Turner, C. L. , Henebry, G. M. , Goodin, D. G. , & Nellis, M. D. (1997). Spatial and temporal patterns of vegetation in the flint hills. Transactions of the Kansas Academy of Science, 100, 10–20. 10.2307/3628435

[ece34394-bib-0501] Brown, K. J. , & Hebda, R. J. (2003). Coastal rainforest connections disclosed through a Late Quaternary vegetation, climate, and fire history investigation from the Mountain Hemlock Zone on southern Vancouver Island, British Colombia, Canada. Review of Palaeobotany and Palynology, 123, 247–269. 10.1016/S0034-6667(02)00195-1

[ece34394-bib-0022] Brown, K. J. , Clark, J. S. , Grimm, E. C. , Donovan, J. J. , Mueller, P. G. , Hansen, B. C. S. , & Stefanova, I. (2005a). Fire cycles in North American interior grasslands and their relation to prairie drought. Proceedings of the National Academy of Sciences of the United States of America, 102, 8865–8870. 10.1073/pnas.0503621102 15956200 PMC1150278

[ece34394-bib-0023] Brown, K. J. , Clark, J. S. , Grimm, E. C. , Donovan, J. J. , Mueller, P. G. , Hansen, B. C. S. , & Stefanova, I. (2005b). Fire cycles in North American interior grasslands and their relation to prairie drought. Proceedings of the National Academy of Sciences of the United States of America, 102, 8865–8870. 10.1073/pnas.0503621102 15956200 PMC1150278

[ece34394-bib-0024] Brown, P. M. , & Sieg, C. H. (1999). Historical variability in fire at the ponderosa pine‐Northern Great Plains prairie ecotone, southeastern Black Hills, South Dakota. Ecoscience, 6, 539–547. 10.1080/11956860.1999.11682563

[ece34394-bib-0025] Brunelle, A. , Minckley, T. A. , & Delgadillo, J. (2015). Using sedimentary records to inform the causes of woody plant encroachment in Southwestern US Desert Grasslands. Quaternary International, 387, 134 10.1016/j.quaint.2015.01.132

[ece34394-bib-0502] Brunelle, A. , Whitlock, C. , Bartlein, P. , & Kipfmueller, K. (2005). Holocene fire and vegetation along environmental gradients in the Northern Rocky Mountains. Quaternary Science Reviews, 24, 2281–2300. 10.1016/j.quascirev.2004.11.010

[ece34394-bib-0026] Camill, P. , Umbanhowar, C. E. , Teed, R. , Geiss, C. E. , Aldinger, J. , Dvorak, L. , … Walkup, K. (2003). Late‐glacial and Holocene climatic effects on fire and vegetation dynamics at the prairie–forest ecotone in south‐central Minnesota. Journal of Ecology, 91, 822–836. 10.1046/j.1365-2745.2003.00812.x

[ece34394-bib-0027] Carcaillet, C. , & Muller, S. D. (2005). Holocene tree limit and distribution of Abies alba in the inner French Alps: Anthropogenic or climatic changes? Boreas, 34, 468–476. 10.1080/03009480500231377

[ece34394-bib-0028] Carrión, J. S. , Sánchez‐Gómez, P. , Mota, J. F. , Yll, R. , & Chaín, C. (2003). Holocene vegetation dynamics, fire and grazing in the Sierra de Gádor, southern Spain. Holocene, 13, 839–849. 10.1191/0959683603hl662rp

[ece34394-bib-0029] Chang Huang, C. , Pang, J. , Chen, S. , Su, H. , Han, J. , Cao, Y. , … Tan, Z. (2006). Charcoal records of fire history in the Holocene loess–soil sequences over the southern Loess Plateau of China. Palaeogeography, Palaeoclimatology, Palaeoecology, 239, 28–44. 10.1016/j.palaeo.2006.01.004

[ece34394-bib-0030] Christensen, L. , Coughenour, M. B. , Ellis, J. E. , & Chen, Z. Z. (2004). Vulnerability of the Asian typical steppe to grazing and climate change. Climatic Change, 63, 351–368. 10.1023/B:CLIM.0000018513.60904.fe

[ece34394-bib-0031] Clark, J. S. , Grimm, E. C. , Donovan, J. J. , Fritz, S. C. , Engstrom, D. R. , & Almendinger, J. E. (2002). Drought cycles and landscape responses to past aridity on prairies of the northern Great Plains, USA. Ecology, 83, 595–601. 10.1890/0012-9658(2002)083[0595:DCALRT]2.0.CO;2

[ece34394-bib-0032] Clark, J. S. , Grimm, E. C. , Lynch, J. , & Mueller, P. G. (2001). Effects of Holocene climate change on the C4 grassland/woodland boundary in the Northern Plains, USA. Ecology, 82, 620–636. 10.1890/0012-9658(2001)082[0620:EOHCCO]2.0.CO;2

[ece34394-bib-0033] Clark, J. S. , & Royall, P. D. (1996). Local and regional sediment charcoal evidence for fire regimes in presettlement north‐eastern North America. Journal of Ecology, 84, 365–382. 10.2307/2261199

[ece34394-bib-0034] Cochrane, M. A. , Alencar, A. , Schulze, M. D. , Souza, C. M. , Nepstad, D. C. , Lefebvre, P. , & Davidson, E. A. (1999). Positive feedbacks in the fire dynamic of closed canopy tropical forests. Science, 284, 1832–1835. 10.1126/science.284.5421.1832 10364555

[ece34394-bib-0035] Colombaroli, D. , Ssemmanda, I. , Gelorini, V. , & Verschuren, D. (2014). Contrasting long‐term records of biomass burning in wet and dry savannas of equatorial East Africa. Global Change Biology, 20, 2903–2914. 10.1111/gcb.12583 24677504

[ece34394-bib-0036] Commerford, J. L. , Grimm, E. C. , Morris, C. J. , Nurse, A. , Stefanova, I. , & McLauchlan, K. K. (2017). Regional variation in Holocene climate quantified from pollen in the Great Plains of North America. International Journal of Climatology, 38, 1794–1807.

[ece34394-bib-0037] Commerford, J. L. , Leys, B. , Mueller, J. R. , & McLauchlan, K. K. (2016). Great Plains vegetation dynamics in response to fire and climatic fluctuations during the Holocene at Fox Lake, Minnesota (USA). Holocene, 26, 10.1177/0959683615608691

[ece34394-bib-0038] Commerford, J. L. , McLauchlan, K. K. , & Sugita, S. (2013). Calibrating vegetation cover and grassland pollen assemblages in the flint hills of Kansas, USA. American Journal of Plant Sciences, 4, 1–10. 10.4236/ajps.2013.47A1001

[ece34394-bib-0039] Conedera, M. , Tinner, W. , Neff, C. , Meurer, M. , Dickens, A. F. , & Krebs, P. (2009). Reconstructing past fire regimes: Methods, applications, and relevance to fire management and conservation. Quaternary Science Reviews, 28, 555–576. 10.1016/j.quascirev.2008.11.005

[ece34394-bib-0040] Craine, J. M. , & McLauchlan, K. K. (2004). The influence of biotic drivers on North American palaeorecords: Alternatives to climate. Holocene, 14(5), 787–791. 10.1191/0959683604hl758fa

[ece34394-bib-0041] Cruz, F. W. , Burns, S. J. , Karmann, I. , Sharp, W. D. , Vuille, M. , Cardoso, A. O. , … Viana, O. (2005). Insolation‐driven changes in atmospheric circulation over the past 116,000 years in subtropical Brazil. Nature, 434, 63–66. 10.1038/nature03365 15744298

[ece34394-bib-0042] Daniau, A. L. , Bartlein, P. J. , Harrison, S. P. , Prentice, I. C. , Brewer, S. , Friedlingstein, P. , … Marlon, J. R. (2012). Predictability of biomass burning in response to climate changes. Global Biogeochemical Cycles, 26, 1–12. 10.1029/2011GB004249

[ece34394-bib-0043] Daniau, A.‐L. , Goñi, M. F. S. , Martinez, P. , Urrego, D. H. , Bout‐Roumazeilles, V. , Desprat, S. , & Marlon, J. R. (2013). Orbital‐scale climate forcing of grassland burning in southern Africa. Proceedings of the National Academy of Sciences, USA, 110, 5069–5073. 10.1073/pnas.1214292110 PMC3612617 23479611

[ece34394-bib-0044] Davis, O. K. , & Shafer, D. S. (2006). Sporormiella fungal spores, a palynological means of detecting herbivore density. Palaeogeography, Palaeoclimatology, Palaeoecology, 237, 40–50. 10.1016/j.palaeo.2005.11.028

[ece34394-bib-0045] DeBano, L. F. (1991). The effect of fire on soil properties. Proceedings‐management Product. West For Soils, 00, 151–156.

[ece34394-bib-0046] Dijkstra, F. A. , Wrage, K. , Hobbie, S. E. , & Reich, P. B. (2006). Tree patches show greater N losses but maintain higher soil N availability than grassland patches in a frequently burned oak savanna. Ecosystems, 9, 441–452. 10.1007/s10021-006-0004-6

[ece34394-bib-0047] Dixon, A. P. , Faber‐Langendoen, D. , Josse, C. , Morrison, J. C. , Loucks, C. J. , Jenkins, C. N. , … Murray, S. (2014). Distribution mapping of world grassland types. Journal of Biogeography, 41, 2003–2019. 10.1126/science.206.4418.550

[ece34394-bib-0048] Duffin, K. I. I. , Gillson, L. , & Willis, K. J. J. (2008). Testing the sensitivity of charcoal as an indicator of fire events in savanna environments: Quantitative predictions of fire proximity, area and intensity. Holocene, 18, 279–291. 10.1177/0959683607086766

[ece34394-bib-0049] Edwards, G. P. , Allan, G. E. , Brock, C. , Duguid, A. , Gabrys, K. , & Vaarzon‐Morel, P. (2008). Fire and its management in central Australia. Rangel Journal, 30, 109–121. 10.1071/RJ07037

[ece34394-bib-0050] Etienne, D. , Wilhelm, B. , Sabatier, P. , Reyss, J.‐L. , & Arnaud, F. (2013). Influence of sample location and livestock numbers on Sporormiella concentrations and accumulation rates in surface sediments of Lake Allos, French Alps. Journal of Paleolimnology, 49, 117–127. 10.1007/s10933-012-9646-x

[ece34394-bib-0051] Fernandes, P. M. , & Botelho, H. S. (2003). A review of prescribed burning effectiveness in fire hazard reduction. International Journal of Wildland Fire, 12, 117–128. 10.1071/WF02042

[ece34394-bib-0052] Feurdean, A. , Liakka, J. , Vannière, B. , Marinova, E. , Hutchinson, S. M. , Mosburgger, V. , & Hickler, T. (2013). 12,000‐Years of fire regime drivers in the lowlands of transylvania (Central‐Eastern Europe): A data‐model approach. Quaternary Science Reviews, 81, 48–61. 10.1016/j.quascirev.2013.09.014

[ece34394-bib-0503] Flood, R.D. , Piper, D. J. W. , Klaus, A. , & Peterson, L.C. (Eds.), (1997). Proceedings of the ocean drilling program: Scientific results, Vol. 155.

[ece34394-bib-0053] Fuhlendorf, S. D. , & Engle, D. M. (2001). Restoring Heterogeneity on Rangelands: Ecosystem Management Based on Evolutionary Grazing Patterns We propose a paradigm that enhances heterogeneity instead of homogeneity to promote biological diversity and wildlife habitat on rangelands grazed by livest. BioScience, 51, 625–632. 10.1641/0006-3568(2001)051[0625:RHOREM]2.0.CO;2

[ece34394-bib-0054] Gavin, D. G. , Hallett, D. J. , Hu, F. S. , Lertzman, K. P. , Prichard, S. J. , Brown, K. J. , … Peterson, D. L. (2007). Forest fire and climate change in western North America: Insights from sediment charcoal records. Frontiers in Ecology and the Environment, 5, 499–506. 10.1890/060161

[ece34394-bib-0055] Genries, A. , Muller, S. D. , Mercier, L. , Bircker, L. , & Carcaillet, C. (2009). Fires control spatial variability of subalpine vegetation dynamics during the Holocene in the Maurienne Valley (French Alps). Ecoscience, 16, 13–22. 10.2980/16-1-3180

[ece34394-bib-0056] Gill, J. L. , McLauchlan, K. K. , Skibbe, A. M. , Goring, S. , Zirbel, C. R. , & Williams, J. W. (2013). Linking abundances of the dung fungus Sporormiella to the density of bison: Implications for assessing grazing by megaherbivores in palaeorecords. Journal of Ecology, 101, 1125–1136. 10.1111/1365-2745.12130

[ece34394-bib-0057] Girardin, M. P. , Ali, A. , Carcaillet, C. , Mudelsee, M. , Drobyshev, I. , Hély, C. , … Christopher, C. (2009). Heterogeneous response of circumboreal wildfire risk to climate change since the early 1900s. Global Change Biology, 15, 2751–2769. 10.1111/j.1365-2486.2009.01869.x

[ece34394-bib-0058] Gradel, A. , Haensch, C. , Ganbaatar, B. , Dovdondemberel, B. , Nadaldorj, O. , & Günther, B. (2017). Response of white birch (*Betula platyphylla* Sukaczev) to temperature and precipitation in the mountain forest steppe and taiga of northern Mongolia. Dendrochronologia, 41, 24–33. 10.1016/j.dendro.2016.03.005

[ece34394-bib-0059] Greenville, A. C. , Dickman, C. R. , Wardle, G. M. , & Letnic, M. (2009). The fire history of an arid grassland: The influence of antecedent rainfall and ENSO. International Journal of Wildland Fire, 18(6), 631–639. 10.1071/WF08093

[ece34394-bib-0060] Grieman, M. M. , Aydin, M. , Isaksson, E. , Schwikowski, M. , & Saltzman, E. S. (2018). Aromatic acids in an Arctic ice core from Svalbard: a proxy record of biomass burning. Climate of the Past, 14(5), 637–651. 10.5194/cp-14-637-2018

[ece34394-bib-0061] Grieman, M. M. , Aydin, M. , McConnell, J. R. , & Saltzman, E. S. (2018). Burning‐derived vanillic acid in an Arctic ice core from Tunu, Northeastern Greenland. Climate of the Past Discussions, 10.5194/cp-2018-46,inreview

[ece34394-bib-0062] Grimm, E. C. , Donovan, J. J. , & Brown, K. J. (2011). A high‐resolution record of climate variability and landscape response from Kettle Lake, northern Great Plains, North America. Quaternary Science Reviews, 30, 2626–2650. 10.1016/j.quascirev.2011.05.015

[ece34394-bib-0063] Grosjean, P. , & Ibanez, F. (2002). Pastecs. Man. l'utilisateur la Libr. Fonct. pour R pour S 290.

[ece34394-bib-0064] Guyette, R. P. , & Stambaugh, M. C. (2004). Post‐oak fire scars as a function of diameter, growth, and tree age. Forest Ecology and Management, 198, 183–192. 10.1016/j.foreco.2004.04.016

[ece34394-bib-0510] Hallett, D. J. , Lepofsky, D. S. , Mathewes, R. W. , & Lertzman, K. P. (2003). 11 000 years of fire history and climate in the mountain hemlock rain forests of southwestern British Columbia based on sedimentary charcoal. Canadian Journal of Forest Research, 33, 292–312. 10.1139/x02-177

[ece34394-bib-0065] Harrison, S. P. , & Bartlein, P. (2012). Records from the past, lessons for the future: what the palaeorecord implies about mechanisms of global change.

[ece34394-bib-0511] Harrison, S. P. , & Prentice, C. I. (2003). Climate and CO2 controls on global vegetation distribution at the last glacial maximum: analysis based on palaeovegetation data, biome modelling and palaeoclimate simulations. Global Change Biology, 9, 983–1004. 10.1046/j.1365-2486.2003.00640.x

[ece34394-bib-0066] Heisler, J. L. , Briggs, J. M. , & Knapp, A. K. (2003). Long‐term patterns of shrub expansion in a C4‐dominated grassland: Fire frequency and the dynamics of shrub cover and abundance. American Journal of Botany, 90(3), 423–428. 10.3732/ajb.90.3.423 21659135

[ece34394-bib-0067] Hély, C. , & Alleaume, S. (2006). Fire regimes in dryland landscapes In D'Odorico P., & Porporato A. (Eds.), Dryland ecohydrology (pp. 283–301). Netherlands: Springer 10.1007/1-4020-4260-4

[ece34394-bib-0068] Hernández, D. L. , & Hobbie, S. E. (2008). Effects of fire frequency on oak litter decomposition and nitrogen dynamics. Oecologia, 158, 535–543. 10.1007/s00442-008-1162-3 18850116

[ece34394-bib-0069] Higuera, P. E. , Sprugel, D. G. , & Brubaker, L. B. (2005). Reconstructing fire regimes with charcoal from small‐hollow sediments: A calibration with tree‐ring records of fire. Holocene, 15, 238–251. 10.1191/0959683605hl789rp

[ece34394-bib-0070] Higuera, P. E. , Whitlock, C. , & Gage, J. A. (2011). Linking tree‐ring and sediment‐charcoal records to reconstruct fire occurrence and area burned in subalpine forests of Yellowstone National Park, USA. Holocene, 21, 327–341. 10.1177/0959683610374882

[ece34394-bib-0071] Hoetzel, S. , Dupont, L. , Schefuß, E. , Rommerskirchen, F. , & Wefer, G. (2013). The role of fire in Miocene to Pliocene C4 grassland and ecosystem evolution. Nature Geoscience, 6, 1027–1030. 10.1038/ngeo1984

[ece34394-bib-0072] Hovmöller, E. (1949). The trough‐and‐ridge diagram. Tellus, 1, 62–66.

[ece34394-bib-0504] Iglesias, V. , & Whitlock, C. (2014). Fire responses to postglacial climate change and human impact in northern Patagonia (41–43 S). Proceedings of the National Academy of Sciences, 111, E5545–E5554. 10.1073/pnas.1410443111 PMC4280613 25489077

[ece34394-bib-0505] Iglesias, V. , Whitlock, C. , Markgraf, V. , & Bianchi, M. M. (2014). Postglacial history of the Patagonian forest/steppe ecotone (41–43 S). Quaternary Science Reviews, 94, 120–135. 10.1016/j.quascirev.2014.04.014

[ece34394-bib-0073] Jacobs, M. J. , & Scholeder, C. A. (2002). Fire frequency and species associations grasslands. African Journal of Ecology, 40, 1 10.1046/j.0141-6707.2001.00347.x

[ece34394-bib-0074] Keeley, J. E. , & Rundel, P. W. (2005). Fire and the Miocene expansion of C4 grasslands. Ecology Letters, 8, 683–690. 10.1111/j.1461-0248.2005.00767.x

[ece34394-bib-0075] Kirchgeorg, T. (2015). Specific molecular markers in lake sediment cores for biomass burning reconstruction during the Holocene. Università Ca'Foscari Venezia.

[ece34394-bib-0076] Knapp, A. K. , Briggs, J. M. , Hartnett, D. C. , & Collins, S. L. (1998). Grassland dynamics: Long‐term ecological research in tallgrass prairie. New York: Oxford University Press.

[ece34394-bib-0506] Lasslop, G. , Brovkin, V. , Reick, C. H. , Bathiany, S. , & Kloster, S. (2016). Multiple stable states of tree cover in a global land surface model due to a fire‐vegetation feedback. Geophysical Research Letters, 43, 6324–6331. 10.1002/2016GL069365

[ece34394-bib-0077] Lehmann, C. E. R. , Anderson, T. M. , Sankaran, M. , Higgins, S. I. , Archibald, S. , Hoffmann, W. A. , … Bond, W. J. (2014). Savanna vegetation‐fire‐climate relationships differ among continents. Science, 343, 548–552. 10.1126/science.1247355 24482480

[ece34394-bib-0507] Lepofsky, D. , Lertzman, K. , Hallett, D. , & Mathewes, R. (2005). Climate change and culture change on the southern coast of British Columbia 2400‐1200 cal. American Antiquity, 70, 267–293.

[ece34394-bib-0078] Levavasseur, G. , Vrac, M. , Roche, D. M. , & Paillard, D. (2012). Statistical modelling of a new global potential vegetation distribution. Environmental Research Letters, 7, 44019 10.1088/1748-9326/7/4/044019

[ece34394-bib-0079] Leys, B. , Brewer, S. C. , McConaghy, S. , Mueller, J. , & McLauchlan, K. K. (2015). Fire history reconstruction in grassland ecosystems: Amount of charcoal reflects local area burned. Environmental Research Letters, 10, 114009 10.1088/1748-9326/10/11/114009

[ece34394-bib-0080] Leys, B. , & Carcaillet, C. (2016). Subalpine fires in the Alps for the past 8000 years: The roles of vegetation, climate and untimely, land uses. Climatic Change, 135, 683–697. 10.1007/s10584-016-1594-4

[ece34394-bib-0081] Leys, B. A. , Commerford, J. L. , & McLauchlan, K. K. (2017). Reconstructing grassland fire history using sedimentary charcoal: Considering count, size and shape. PLoS ONE, 12, e0176445 10.1371/journal.pone.0176445 28448597 PMC5407794

[ece34394-bib-0082] Lynch, J. A. , Clark, J. S. , & Stocks, B. J. (2004). Charcoal production, dispersal, and deposition from the Fort Providence experimental fire: Interpreting fire regimes from charcoal records in boreal forests. Canadian Journal of Forest Research, 34, 1642–1656. 10.1139/x04-071

[ece34394-bib-0083] Marlon, J. R. , Bartlein, P. J. , Carcaillet, C. , Gavin, D. G. G. , Harrison, S. P. , Higuera, P. E. , … Prentice, I. C. C. (2008). Climate and human influences on global biomass burning over the past two millennia. Nature Geoscience, 1, 697–702. 10.1038/ngeo468

[ece34394-bib-0084] Marlon, J. R. , Bartlein, P. J. , Daniau, A.‐L. , Harrison, S. P. , Maezumi, S. Y. , Power, M. J. , … Vanniere, B. (2013). Global biomass burning: A synthesis and review of Holocene paleofire records and their controls. Quaternary Science Reviews, 65, 5–25. 10.1016/j.quascirev.2012.11.029

[ece34394-bib-0085] Marlon, J. , Bartlein, P. J. , & Whitlock, C. (2006). Fire‐fuel‐climate linkages in the northwestern USA during the Holocene. Holocene, 16, 1059–1071. 10.1177/0959683606069396

[ece34394-bib-0086] Marlon, J. R. , Kelly, R. , Daniau, A.‐L. , Vannière, B. , Power, M. J. , Bartlein, P. , … Brücher, T. (2016). Reconstructions of biomass burning from sediment‐charcoal records to improve data–model comparisons. Biogeosciences, 13, 3225–3244. 10.5194/bg-13-3225-2016

[ece34394-bib-0087] McLauchlan, K. K. , Commerford, J. L. , & Morris, C. J. (2013). Tallgrass prairie pollen assemblages in mid‐continental North America. Vegetation History and Archaeobotany, 22, 171–183. 10.1007/s00334-012-0369-8

[ece34394-bib-0512] McWethy, D. B. , Whitlock, C. , Wilmshurst, J. M. , McGlone, M. S. , & Li, X. (2009). Rapid deforestation of South Island, New Zealand, by early Polynesian fires. The Holocene, 19, 883–897. 10.1177/0959683609336563

[ece34394-bib-0513] Millspaugh, S. H. , Whitlock, C. , & Bartlein, P. J. (2000). Variations in fire frequency and climate over the past 17 000 yr in central Yellowstone National Park. Geology, 28, 211–214. 10.1130/0091-7613(2000)28<211:VIFFAC>2.0.CO;2

[ece34394-bib-0088] Mooney, S. D. , Harrison, S. P. , Bartlein, P. J. , Daniau, A.‐L. , Stevenson, J. , Brownlie, K. C. , … Black, M. (2011). Late Quaternary fire regimes of Australasia. Quaternary Science Reviews, 30, 28–46. 10.1016/j.quascirev.2010.10.010

[ece34394-bib-0514] Moreno, P. I. (2000). Climate, fire, and vegetation between about 13,000 and 9200 14 C yr BP in the Chilean Lake District. Quaternary Research, 54, 81–89. 10.1006/qres.2000.2148

[ece34394-bib-0089] Morgan, J. W. (1999). Defining grassland fire events and the response of perennial plants to annual fire in temperate grasslands of south‐eastern Australia. Plant Ecology, 144(1), 127–144. 10.1023/A:1009731815511

[ece34394-bib-0090] Mouillot, F. , & Field, C. B. (2005). Fire history and the global carbon budget: A 1ox 1o fire history reconstruction for the 20th century. Global Change Biology, 11, 398–420. 10.1111/j.1365-2486.2005.00920.x

[ece34394-bib-0091] Murray, S. , White, R. , & Rohweder, M. (2000). Pilot Analysis of Global Ecosystems: Grasslands Ecosystems.

[ece34394-bib-0092] Nayak, R. R. , Vaidyanathan, S. , & Krishnaswamy, J. (2014). Fire and grazing modify grass community response to environmental determinants in savannas: Implications for sustainable use. Agriculture, Ecosystems & Environment, 185, 197–207. 10.1016/j.agee.2014.01.002

[ece34394-bib-0093] Neary, D. G. , Klopatek, C. C. , DeBano, L. F. , & Ffolliott, P. F. (1999). Fire effects on belowground sustainability: A review and synthesis. Forest Ecology and Management, 122, 51–71. 10.1016/S0378-1127(99)00032-8

[ece34394-bib-0094] Nelson, D. M. , Hu, F. S. , Grimm, E. C. , Curry, B. B. , Slate, J. E. , Feng, S. H. , … Slate, J. E. (2006). The influence of aridity and fire on Holocene prairie communities in the eastern Prairie Peninsula. Ecology, 87, 2523–2536. 10.1890/0012-9658(2006)87[2523:TIOAAF]2.0.CO;2 17089661

[ece34394-bib-0095] Nelson, D. M. , Hu, F. S. , Tian, J. , Stefanova, I. , & Brown, T. A. (2004). Response of C3 and C4 plants to middle‐Holocene climatic variation near the prairie–forest ecotone of Minnesota. Proceedings of the National Academy of Sciences of the United States of America, 101, 562–567. 10.1073/pnas.0307450100 14701908 PMC327187

[ece34394-bib-0096] Nelson, D. M. , Verschuren, D. , Urban, M. A. , & Hu, F. S. (2012). Long‐term variability and rainfall control of savanna fire regimes in equatorial East Africa. Global Change Biology, 18, 3160–3170. 10.1111/j.1365-2486.2012.02766.x 28741834

[ece34394-bib-0097] Nepstad, D. C. , Verssimo, A. , Alencar, A. , Nobre, C. , Lima, E. , Lefebvre, P. , … Mendoza, E. (1999). Large‐scale impoverishment of Amazonian forests by logging and fire. Nature, 398, 505–508. 10.1038/19066

[ece34394-bib-0098] Ohrtman, M. K. , Clay, S. A. , & Smart, A. J. (2015). Surface temperatures and durations associated with spring prescribed fires in eastern South Dakota tallgrass prairies. American Midland Naturalist, 173, 88–98. 10.1674/0003-0031-173.1.88

[ece34394-bib-0099] Oyarzabal, M. , Paruelo, J.M. , Federico, P. , Oesterheld, M. , & Lauenroth, W. K. (2008). Trait differences between grass species along a climatic gradient in South and North America. Journal of Vegetation Science, 19, 183‐U1 https://doi.org/doi 10.3170/2007‐8‐18349

[ece34394-bib-0100] Pastro, L. A. , Dickman, C. R. , & Letnic, M. (2011). Burning for biodiversity or burning biodiversity? Prescribed burn vs. wildfire impacts on plants, lizards, and mammals. Ecological Applications, 21, 3238–3253. 10.1890/10-2351.1

[ece34394-bib-0101] Patterson, W. A. , Edwards, K. J. , & Maguire, D. J. (1987). Microscopic charcoal as a fossil indicator of fire. Quaternary Science Reviews, 6, 3–23. 10.1016/0277-3791(87)90012-6

[ece34394-bib-0102] Pellegrini, A. F. , Ahlström, A. , Hobbie, S. E. , Reich, P. B. , Nieradzik, L. P. , Staver, A. C. , … Jackson, R. B. (2018). Fire frequency drives decadal changes in soil carbon and nitrogen and ecosystem productivity. Nature, 553(7687), 194. 29227988 10.1038/nature24668

[ece34394-bib-0104] Pitkanen, A. , Lehtonen, H. , & Huttunen, P. (1999). Comparison of sedimentary microscopic charcoal particle records in a small lake with dendrochronological data: Evidence for the local origin of microscopic charcoal produced by forest fires of low intensity in eastern Finland. Holocene, 9, 559–567. 10.1191/095968399670319510

[ece34394-bib-0515] Power, M. J. , Mayle, F. E. , Bartlein, P. J. , Marlon, J. R. , Anderson, R. S. , Behling, H. , Brown, K. J. , Carcaillet, C. , Colombaroli, D. , & Gavin, D. G. (2013). Climatic control of the biomass‐burning decline in the Americas after AD 1500. The Holocene, 23, 3–13. 10.1177/0959683612450196

[ece34394-bib-0105] Power, M. J. , Marlon, J. R. , Bartlein, P. J. , & Harrison, S. P. (2010). Fire history and the Global Charcoal Database: A new tool for hypothesis testing and data exploration. Palaeogeography, Palaeoclimatology, Palaeoecology, 291, 52–59. 10.1016/j.palaeo.2009.09.014

[ece34394-bib-0106] Power, M. J. , Marlon, J. , Ortiz, N. , Bartlein, P. J. , Harrison, S. P. , Mayle, F. E. , … Zhang, J. H. (2008). Changes in fire regimes since the Last Glacial Maximum: An assessment based on a global synthesis and analysis of charcoal data. Climate Dynamics, 30, 887–907. 10.1007/s00382-007-0334-x

[ece34394-bib-0107] R Core Team (2014). R: A language and environment for statistical computing. Vienna, Austria: R Foundation for Statistical Computing.

[ece34394-bib-0108] Rabin, S. S. , Melton, J. R. , Lasslop, G. , Bachelet, D. , Forrest, M. , Hantson, S. , … Chao, Y. (2017). The Fire Modeling Intercomparison Project (FireMIP), phase 1: Experimental and analytical protocols with detailed model descriptions. Geoscientific Model Development, 10(3), 1175 10.5194/gmd-10-1175-2017

[ece34394-bib-0109] Ramankutty, N. , & Foley, J. A. (1999). Estimating historical changes in global land cover: Croplands from 1700 to 1992. Global Biogeochemical Cycles, 13, 997–1027. 10.1029/1999GB900046

[ece34394-bib-0110] Raper, D. , & Bush, M. (2009). A test of Sporormiella representation as a predictor of megaherbivore presence and abundance. Quaternary Research, 71, 490–496. 10.1016/j.yqres.2009.01.010

[ece34394-bib-0111] Reich, P. B. , Peterson, D. W. , Wedin, D. A. , & Wrage, K. (2001). Fire and vegetation effects on productivity and nitrogen cycling across a forest‐grassland continuum. Ecology, 82, 1703–1719. 10.2307/2679812

[ece34394-bib-0112] Ripley, B. , Visser, V. , Christin, P.‐A. , Archibald, S. , Martin, T. , & Osborne, C. (2015). Fire ecology of C3 and C4 grasses depends on evolutionary history and frequency of burning but not photosynthetic type. Ecology, 96, 2679–2691. 10.1890/14-1495.1 26649389

[ece34394-bib-0113] Robin, V. , Nelle, O. , Talon, B. , Poschlod, P. , Schwartz, D. , Bal, M. C. , … Dutoit, T. (2018). A comparative review of soil charcoal data: Spatiotemporal patterns of origin and long‐term dynamics of Western European nutrient‐poor grasslands. Holocene, 0959683618771496

[ece34394-bib-0516] Roos, K. , Rollenbeck, R. , Peters, T. , Bendix, J. , & Beck, E. (2010). Growth of tropical bracken (Pteridium arachnoideum): response to weather variations and burning. Invasive Plant Science and Management, 3, 402–411. 10.1614/IPSM-D-09-00031.1

[ece34394-bib-0114] Scasta, J. D. , Duchardt, C. , Engle, D. M. , Miller, J. R. , Debinski, D. M. , & Harr, R. N. (2016). Constraints to restoring fire and grazing ecological processes to optimize grassland vegetation structural diversity. Ecological Engineering, 95, 865–875. 10.1016/j.ecoleng.2016.06.096

[ece34394-bib-0517] Scharf, E. A. (2010). A statistical evaluation of the relative influences of climate, vegetation, and prehistoric human population on the charcoal record of Five Lakes, Washington (USA). Quaternary International, 215, 74–86. 10.1016/j.quaint.2009.09.021

[ece34394-bib-0115] Scheiter, S. , Higgins, S. I. , Osborne, C. P. , Bradshaw, C. , Lunt, D. , Ripley, B. S. , … Beerling, D. J. (2012). Fire and fire‐adapted vegetation promoted C 4 expansion in the late Miocene. New Phytologist, 195, 653–666. 10.1111/j.1469-8137.2012.04202.x 22712748

[ece34394-bib-0116] Scholes, R. J. , & Hall, D. O. (1996). The carbon budget of tropical savannas, woodlands and grasslands. Scientific Committee on Problems of the Environment, 56, 69–100.

[ece34394-bib-0117] Scurlock, J. M. O. , & Hall, D. O. (1998). The global carbon sink: A grassland perspective. Global Change Biology, 4, 229–233. 10.1046/j.1365-2486.1998.00151.x

[ece34394-bib-0118] Shinoda, M. , Ito, S. , Nachinshonhor, G. U. , & Erdenetsetseg, D. (2007). Phenology of Mongolian grasslands and moisture conditions. Journal of the Meteorological Society of Japan, 85, 359–367. 10.2151/jmsj.85.359

[ece34394-bib-0119] Shuman, B. N. , & Marsicek, J. (2016). The structure of Holocene climate change in mid‐latitude North America. Quaternary Science Reviews, 141, 38–51. 10.1016/j.quascirev.2016.03.009

[ece34394-bib-0120] Simoneit, B. R. (2002). Biomass burning—a review of organic tracers for smoke from incomplete combustion. Applied Geochemistry, 17(3), 129–162. 10.1016/S0883-2927(01)00061-0

[ece34394-bib-0121] Staver, A. C. , Archibald, S. , & Levin, S. A. (2011). The global extent and determinants of savanna and forest as alternative biome states. Science, 334, 230–232. 10.1126/science.1210465 21998389

[ece34394-bib-0122] Still, C.J. , Berry, J.A. , Collatz, G.J. , & DeFries, R.S. (2003). Global distribution of C 3 and C 4 vegetation: Carbon cycle implications. Global Biogeochemical Cycles, 17, 6‐1‐6‐14 10.1029/2001gb001807

[ece34394-bib-0123] Sugita, S. (1993). A model of pollen source area for an entire lake surface. Quaternary Research, 39, 239–244. 10.1006/qres.1993.1027

[ece34394-bib-0124] Sugita, S. (1994). Pollen representation of vegetation in Quaternary sediments: Theory and method in patchy vegetation. Journal of Ecology, 82, 881–897. 10.2307/2261452

[ece34394-bib-0125] Swetnam, T. W. , Allen, C. D. , & Betancourt, J. L. (1999). Applied historical ecology: using the past to manage for the future. Ecological Applications, 9, 1189–1206. 10.1890/1051-0761(1999)009[1189:AHEUTP]2.0.CO;2

[ece34394-bib-0126] Swetnam, T. W. , & Betancourt, J. L. (1990). Fire‐southern oscillation relations in the southwestern United States. Science(Washington), 249, 1017–1020. 10.1126/science.249.4972.1017 17789609

[ece34394-bib-0127] Swetnam, T. W. , & Betancourt, J. L. (1998). Mesoscale disturbance and ecological response to decadal climatic variability in the American Southwest. Journal of Climate, 11, 3128–3147. 10.1175/1520-0442(1998)011<3128:MDAERT>2.0.CO;2

[ece34394-bib-0521] Tarasov, P. , Bezrukova, E. , Karabanov, E. , Nakagawa, T. , Wagner, M. , Kulagina, N. , Letunova, P. , Abzaeva, A. , Granoszewski, W. , & Riedel, F. (2007). Vegetation and climate dynamics during the Holocene and Eemian interglacials derived from Lake Baikal pollen records. Palaeogeography, Palaeoclimatology, Palaeoecology, 252, 440–457. 10.1016/j.palaeo.2007.05.002

[ece34394-bib-0128] Tauber, H. (1974). A static non‐overload pollen collector. New Phytologist, 73, 359–369. 10.1111/j.1469-8137.1974.tb04770.x

[ece34394-bib-0129] Tierney, J. E. , & deMenocal, P. B. (2013). Abrupt shifts in horn of Africa hydroclimate since the last glacial maximum. Science, 342, 843–846. 10.1126/science.1240411 24114782

[ece34394-bib-0130] Tinner, W. , Ammann, B. , & Germann, P. (1996). Treeline fluctuations recorded for 12,500 years by soil profiles, pollen, and plant macrofossils in the Central Swiss Alps. Arctic and Alpine Research, 28, 131–147. 10.2307/1551753

[ece34394-bib-0522] Turner, R. , Roberts, N. , & Jones, M. D. (2008). Climatic pacing of Mediterranean fire histories from lake sedimentary microcharcoal. Global and Planetary Change, 63, 317–324. 10.1016/j.gloplacha.2008.07.002

[ece34394-bib-0131] Umbanhowar, C. E. (2004). Interactions of climate and fire at two sites in the northern Great Plains, USA. Palaeogeography Palaeoclimatology Palaeoecology, 208, 141–152. 10.1016/j.palaeo.2004.03.001

[ece34394-bib-0132] Umbanhowar, C. E. , & Mcgrath, M. J. (1998). Experimental production and analysis of microscopic charcoal from wood, leaves and grasses. Holocene, 8, 341–346. 10.1191/095968398666496051

[ece34394-bib-0133] Umbanhowar, C. E. , Shinneman, L. C. , Tserenkhand, G. , Hammon, E. R. , Lor, P. , & Nail, K. (2009). Regional fire history based on harcoal analysis of sediments from nine lakes in western Mongolia. Holocene, 19, 611–624. 10.1177/0959683609104039

[ece34394-bib-0134] Valkó, O. , Török, P. , Deák, B. , & Tóthmérész, B. (2014). Review: Prospects and limitations of prescribed burning as a management tool in European grasslands. Basic and Applied Ecology, 15, 26–33. 10.1016/j.baae.2013.11.002

[ece34394-bib-1001] Vannière, B. , Power, M. J. , Roberts, N. , Tinner, W. , Carrion, J. , Magny, M. , Bartlein, P. , Colombaroli, D. , Daniau, A. L. , & Finsinger, W. (2011). Circum‐Mediterranean fire activity and climate changes during the mid‐Holocene environmental transition (8500‐2500 cal. BP). The Holocene, 21, 53–73. 10.1177/0959683610384164

[ece34394-bib-0135] Vannière, B. , Blarquez, O. , Rius, D. , Doyen, E. , Brücher, T. , Colombaroli, D. , … Olofsson, J. (2016). 7000‐year human legacy of elevation‐dependent European fire regimes. Quaternary Science Reviews, 132, 206–212. 10.1016/j.quascirev.2015.11.012

[ece34394-bib-0136] Veblen, T. T. , Kitzberger, T. , Villalba, R. , & Donnegan, J. (1999). Fire history in northern Patagonia: The roles of humans and climatic variation. Ecological Monographs, 69(1), 47–67. 10.1890/0012-9615(1999)069[0047:FHINPT]2.0.CO;2

[ece34394-bib-0137] Veblen, T. T. , & Lorenz, D. C. (1988). Recent vegetation changes along the forest/steppe ecotone of northern Patagonia. Annals of the Association of American Geographers, 78, 93–111. 10.1111/j.1467-8306.1988.tb00193.x

[ece34394-bib-0138] Veldman, J. W. , Brudvig, L. A. , Damschen, E. I. , Orrock, J. L. , Mattingly, W. B. , & Walker, J. L. (2014). Fire frequency, agricultural history and the multivariate control of pine savanna understorey plant diversity. Journal of Vegetation Science, 25, 1438–1449. 10.1111/jvs.12195

[ece34394-bib-0139] Veldman, J. W. , Buisson, E. , Durigan, G. , Fernandes, G. W. , Le Stradic, S. , Mahy, G. , … Bond, W. J. (2015). Toward an old‐growth concept for grasslands, savannas, and woodlands. Frontiers in Ecology and the Environment, 13, 154–162. 10.1890/140270

[ece34394-bib-0518] Walsh, M. K. , Whitlock, C. , & Bartlein, P. J. (2008). A 14,300‐year‐long record of fire–vegetation–climate linkages at Battle Ground Lake, southwestern Washington. Quaternary Research, 70, 251–264. 10.1016/j.yqres.2008.05.002

[ece34394-bib-0519] Walsh, M. K. , Marlon, J. R. , Goring, S. J. , Brown, K. J. , & Gavin, D. G. (2015). A regional perspective on Holocene fire–climate–human interactions in the Pacific Northwest of North America. Annals of the Association of American Geographers, 105, 1135–1157. 10.1080/00045608.2015.1064457

[ece34394-bib-0140] Wang, X. , & Li, A. (2007). Preservation of black carbon in the shelf sediments of the East China Sea. Chinese Science Bulletin, 52, 3155–3161. 10.1007/s11434-007-0452-1

[ece34394-bib-0141] van der Werf, G. R. , Randerson, J. T. , Giglio, L. , Collatz, G. J. , Kasibhatla, P. S. , & Arellano, A. F. Jr (2006). Interannual variability in global biomass burning emissions from 1997 to 2004. Atmospheric Chemistry and Physics, 6, 3423–3441. 10.5194/acp-6-3423-2006

[ece34394-bib-0142] Whelan, R.J. (1995). . The ecology of fire, Cambridge, UK: Cambridge University Press.

[ece34394-bib-0143] White, R. P. , Murray, S. , Rohweder, M. , Prince, S. D. , & Thompson, K. M. J. (2000). Grassland ecosystems. Washington, DC: World Resources Institute.

[ece34394-bib-0520] Whitlock, C. , Moreno, P. I. , & Bartlein, P. (2007). Climatic controls of Holocene fire patterns in southern South America. Quaternary Research, 68, 28–36. 10.1016/j.yqres.2007.01.012

[ece34394-bib-0144] Whitlock, C. , & Anderson, R.S. (2003). Fire history reconstructions based on sediment records from lakes and wetlands In: (Eds.), Fire and climatic change in temperate ecosystems of the western Americas (pp. 3–31).: Springer 10.1007/b97443

[ece34394-bib-0145] Whitlock, C. , Bianchi, M. M. , Bartlein, P. J. , Markgraf, V. , Marlon, J. , Walsh, M. , & McCoy, N. (2006). Postglacial vegetation, climate, and fire history along the east side of the Andes (lat 41–42.5 S), Argentina. Quaternary Research, 66, 187–201. 10.1016/j.yqres.2006.04.004

[ece34394-bib-0146] Whitlock, C. , Marlon, J. , Briles, C. , Brunelle, A. , Long, C. , & Bartlein, P. (2008). Long‐term relations among fire, fuel, and climate in the north‐western US based on lake‐sediment studies. International Journal of Wildland Fire, 17, 72–83. 10.1071/WF07025

[ece34394-bib-0147] Willerslev, E. , & Cooper, A. (2005). Review paper ancient DNA. Proceedings of the Royal Society B: Biological Sciences, 272, 3–16. 10.1098/rspb.2004.2813 15875564 PMC1634942

[ece34394-bib-0148] Williams, J. W. , & Shuman, B. (2008). Obtaining accurate and precise environmental reconstructions from the modern analog technique and North American surface pollen dataset. Quaternary Science Reviews, 27, 669–687. 10.1016/j.quascirev.2008.01.004

[ece34394-bib-0149] Zhijun, T. , Jiquan, Z. , & Xingpeng, L. (2009). GIS‐based risk assessment of grassland fire disaster in western Jilin province, China. Stochastic Environmental Research and Risk Assessment, 23(4), 463–471. 10.1007/s00477-008-0233-7

