# Supplementary material for: Global fire history of grassland biomes
Source: Ecol Evol. 2018 Aug 10;8(17):8831–52. doi: 10.1002/ece3.4394 (PMC6157676; doi:10.1002/ece3.4394)
Supplement: Supplementary file 1 [file ECE3-8-8831-s001.pdf]

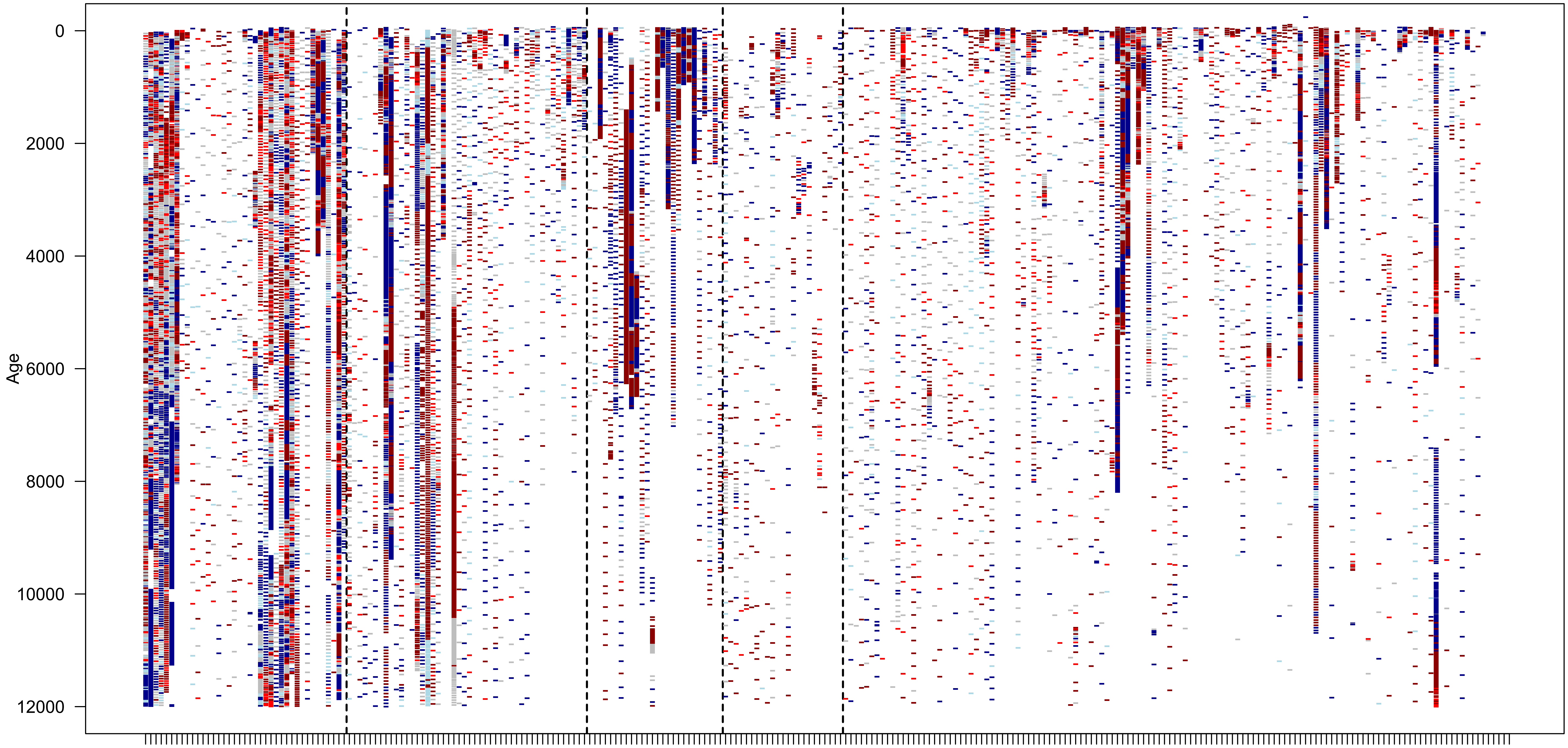

Fig S1: Hovmöller-type diagram with Z-scores of transformed charcoal records from the 262-selected series corresponding to grassland biomes following Levavasseur et al. 2012 (L12). Tick marks represent individual samples with colours underlining periods with dominant positive (pink) or negative (blue) Z-score values.
