# Supplementary material for: Global fire history of grassland biomes
Source: Ecol Evol. 2018 Aug 10;8(17):8831–52. doi: 10.1002/ece3.4394 (PMC6157676; doi:10.1002/ece3.4394)
Supplement: Supplementary file 2 [file ECE3-8-8831-s002.pdf]

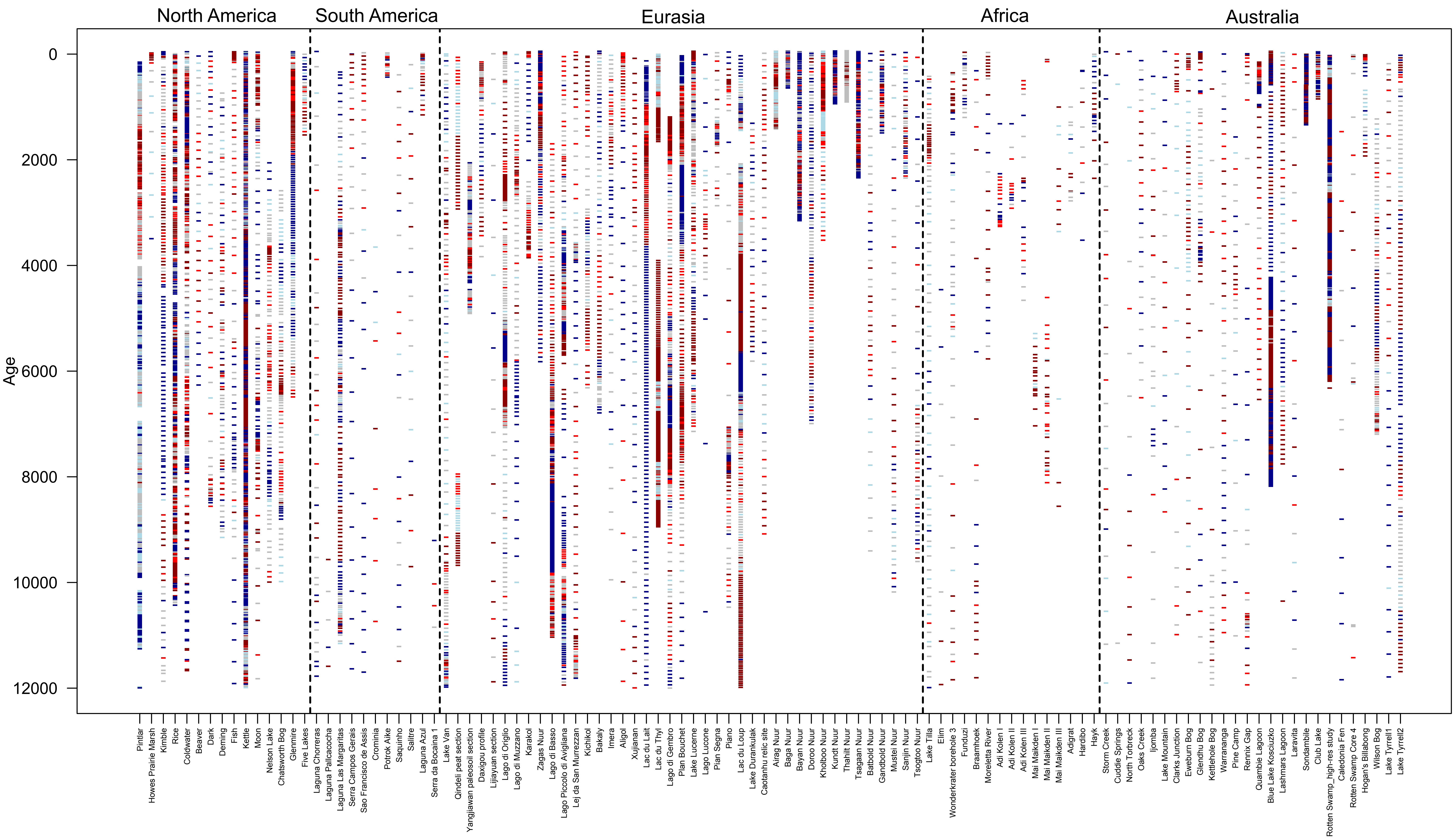

Fig S2: Hovmöller-type diagram with Z-scores of transformed charcoal records from the 108-selected series corresponding to sites overlapping current grassland distribution published by Dixon et al. 2014 (D14). Tick marks represent individual samples with colours underlining periods with dominant positive (pink) or negative (blue) Z-score values.
