# Supplementary material for: Global fire history of grassland biomes
Source: Ecol Evol. 2018 Aug 10;8(17):8831–52. doi: 10.1002/ece3.4394 (PMC6157676; doi:10.1002/ece3.4394)
Supplement: Supplementary file 3 [file ECE3-8-8831-s003.pdf]

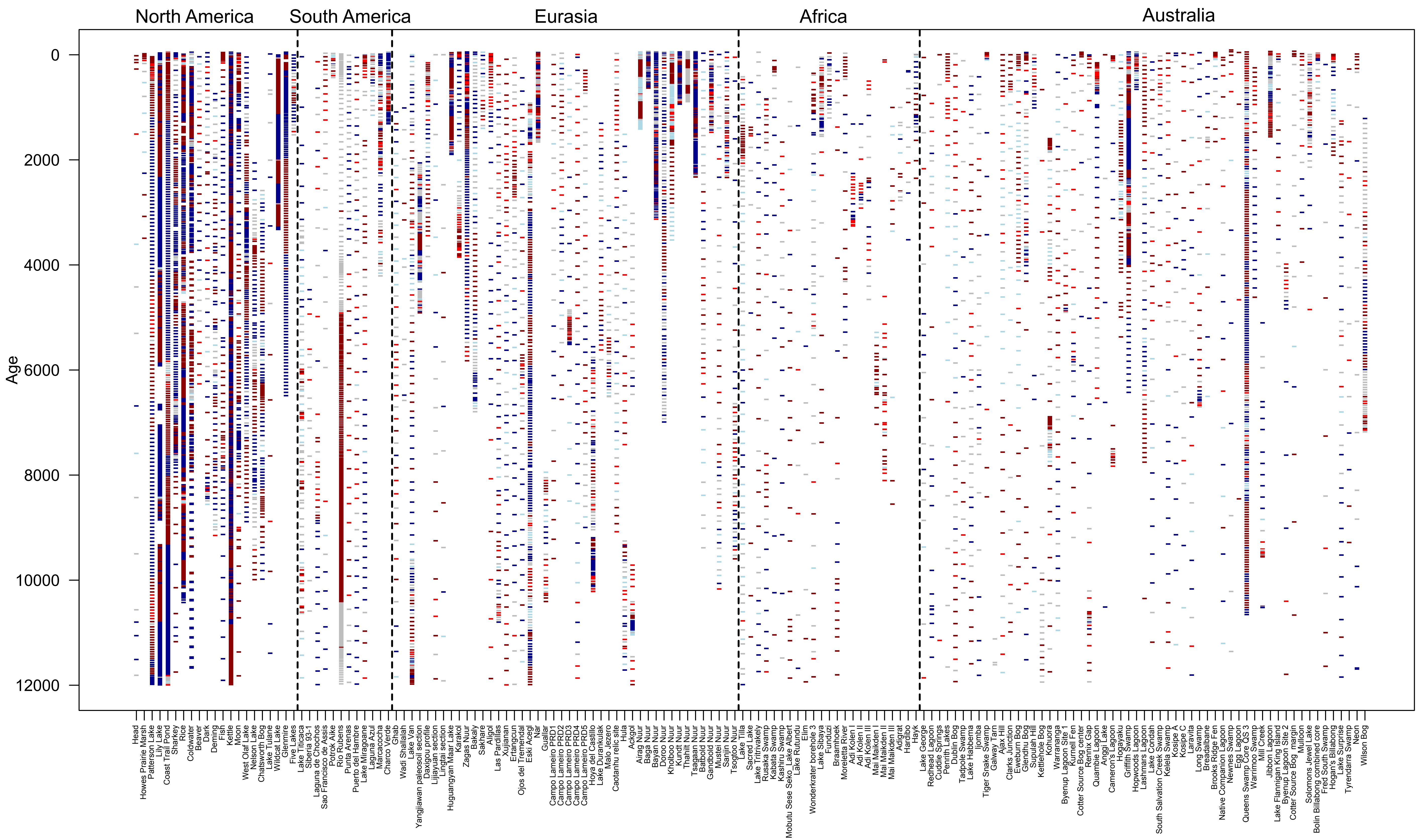

Fig S3: Hovmöller-type diagram with Z-scores of transformed charcoal records from the 157-selected series corresponding to grassland biomes following Ramankatty and Foley 1999 (RF99). Tick marks represent individual samples with colours underlining periods with dominant positive (pink) or negative (blue) Z-score values.
