# Supplementary material for: Global fire history of grassland biomes
Source: Ecol Evol. 2018 Aug 10;8(17):8831–52. doi: 10.1002/ece3.4394 (PMC6157676; doi:10.1002/ece3.4394)
Supplement: Supplementary file 4 [file ECE3-8-8831-s004.pdf]

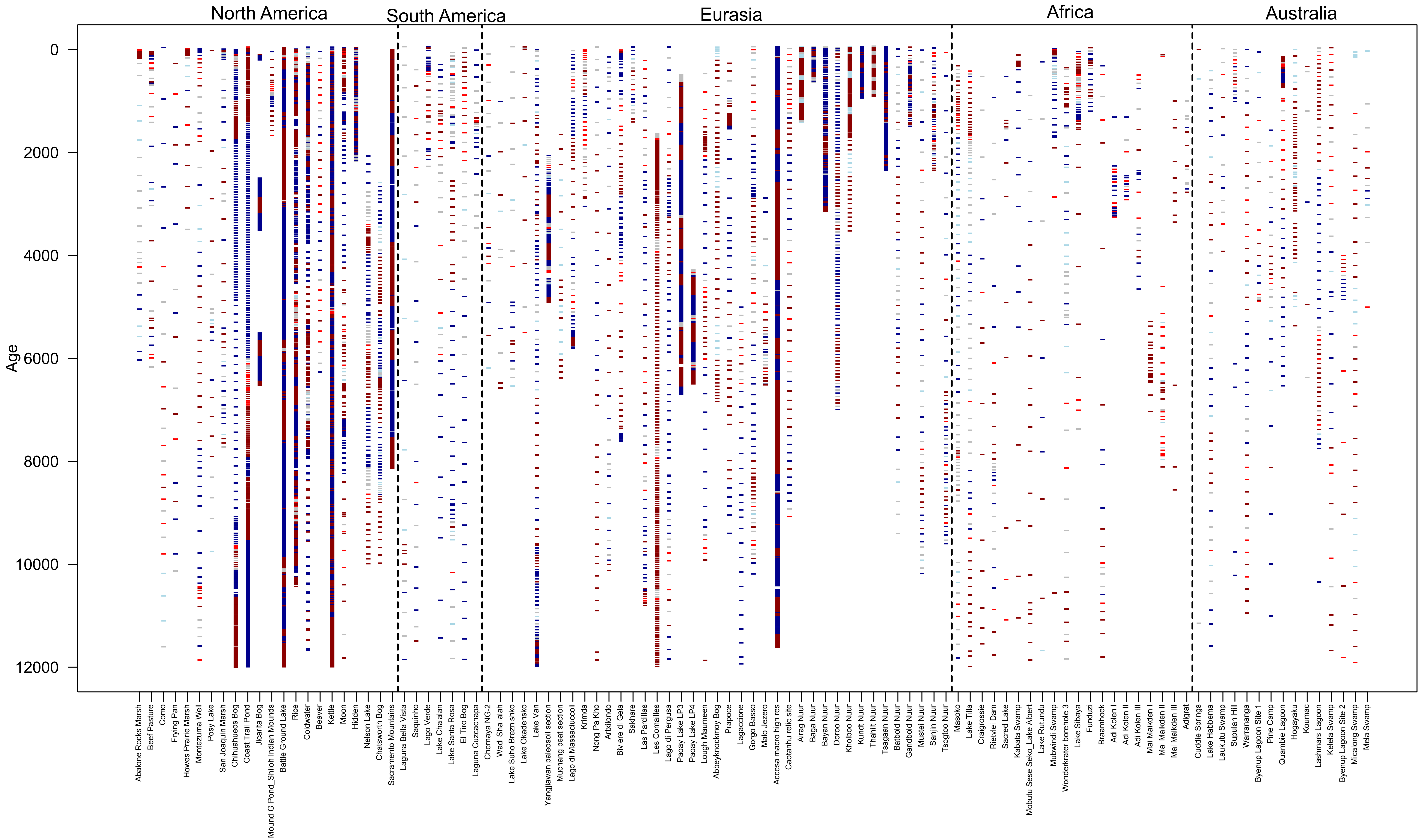

Fig S4: Hovmöller-type diagram with Z-scores of transformed charcoal records from the 103-selected series corresponding to grassland biomes following the Global Charcoal Database contributors (GCD). Tick marks represent individual samples with colours underlining periods with dominant positive (pink) or negative (blue) Z-score values.
