# Supplementary material for: Global fire history of grassland biomes
Source: Ecol Evol. 2018 Aug 10;8(17):8831–52. doi: 10.1002/ece3.4394 (PMC6157676; doi:10.1002/ece3.4394)
Supplement: Supplementary file 5 [file ECE3-8-8831-s005.pdf]

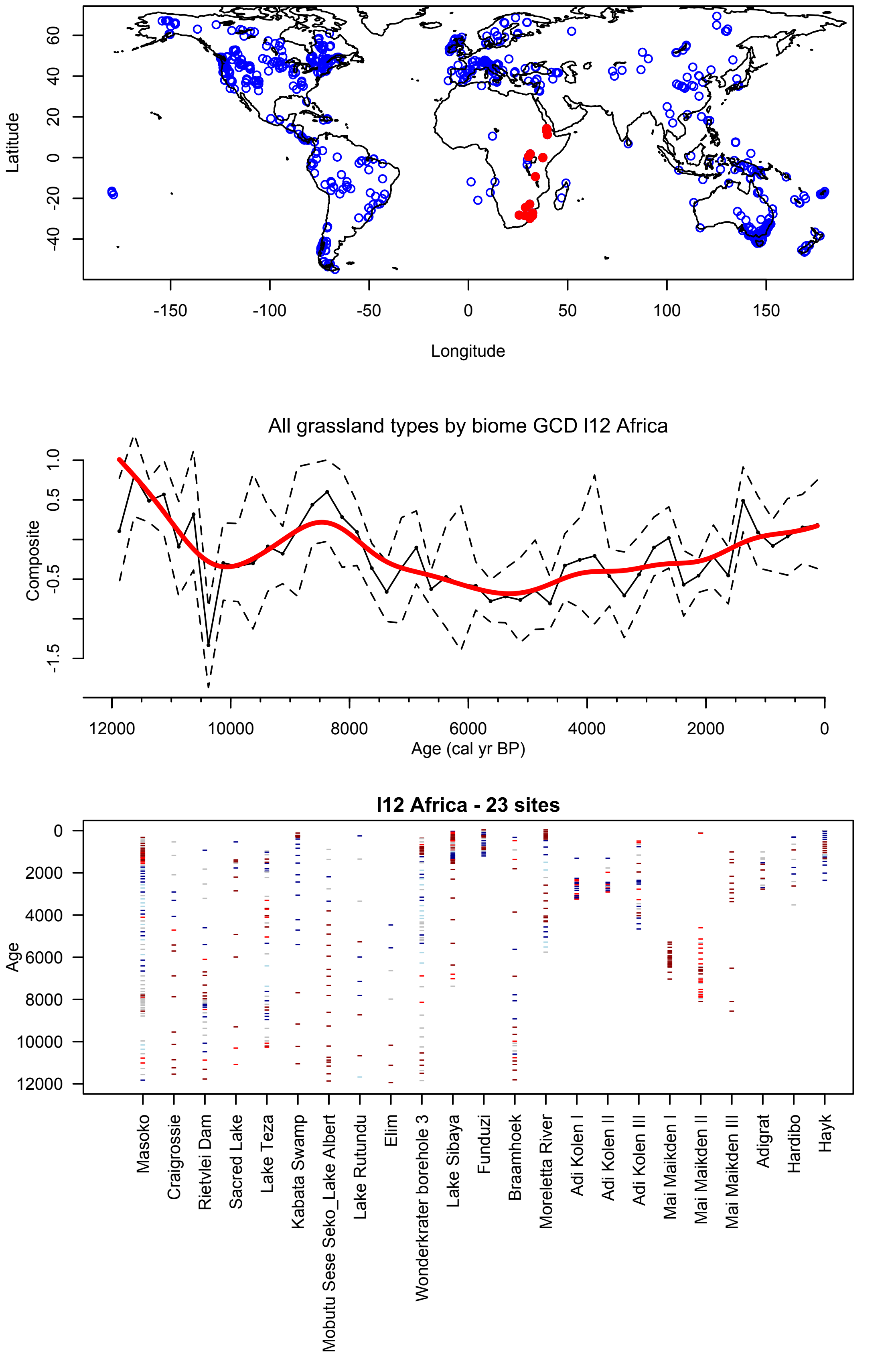

Fig S5: Summary of the charcoal signal of grassland sites following Levavasseur et al. (2012) classification (L12) in Africa. A. Distribution map of the selected sites in red, compare to all the sites in the GCD in blue. B. Normalized charcoal signal of the selected charcoal records on the last 12,000 years. Charcoal records have been standardized (min max method), and normalized (Box-Cox and Z-score transformations). The red lines represent the normalized charcoal data smoothed at 250-year windows, and the dashed lines represent the 95% confident intervals. C. Hovmöller-type diagram with Z-scores of transformed charcoal records from the 23-selected series. Tick marks represent individual samples with colours underlining periods with dominant positive (pink) or negative (blue) Z-score values.
